# Supplementary material for: Facet effect of hematite on the hydrolysis of phthalate esters under ambient humidity conditions
Source: Nat Commun. 2022 Oct 17;13:6125. doi: 10.1038/s41467-022-33950-1 (PMC9576771; doi:10.1038/s41467-022-33950-1)
Supplement: Supplementary file 1 — Supplementary Information [file 41467_2022_33950_MOESM1_ESM.pdf]

Supplementary Information

Facet effect of hematite on the hydrolysis of phthalate esters under ambient humidity conditions

Xin Jin [1], Dingding Wu [1], Cun Liu [2], Shuhan Huang [1], Ziyan Zhou [1], Hao Wu [1], Xiru Chen [1], Meiying Huang [2], Shaoda Zhou [3], Cheng Gu [1,*]

[1] State Key Laboratory of Pollution Control and Resource Reuse, School of the Environment, Nanjing University, Nanjing 210023, China

[2] Institute of Soil Science, Chinese Academy of Sciences, Nanjing 210008, China

[3] Nanjing Kaver Scientific Instrument Co. Ltd., Nanjing 210042, China

* Corresponding author: chenggu@nju.edu.cn

Post address: 163 Xianlin Avenue, Qixia District, Nanjing, 210023, Jiangsu, China.

Tel/fax: +86-025-89680595;

This supporting information contains 2 Supplementary Notes, 5 Supplementary Methods, 4 Supplementary Tables, and 26 Supplementary Figures.

Supplementary Notes

Supplementary Note **1**. Characterizations about surface carbon residual.

Supplementary Note **2**. Calculations of the single Fe site density (D_{si}) and the "probability bidentate Fe-Fe site density" ($D_{p\text{-}bi}$).

Supplementary Methods

Supplementary Method **1**. Chemicals and reagents.

Supplementary Method **2**. Methods for synthesizing HNP/HNR/HNC.

Supplementary Method **3**. Characterization methods.

Supplementary Method **4**. High performance liquid chromatography (HPLC) analytical methods.

Supplementary Method **5**. Gas chromatography mass spectrometer (GC-MS) analytical method.

Supplementary Tables

Supplementary Table **1**. Surface energies (γ) for individual facet termination.

Supplementary Table **2**. The BET-SSA normalized hydrolysis rate constants of DMP and DnBP on HNP, HNR and HNC, in proportional to the surface site density.

Supplementary Table **3**. The calculated turnover number (TON) and turnover frequency (TOF) values of the HNP, HNR and HNC for the hydrolytic degradation of DMP, DnBP and MB under RH 76%.

Supplementary Table **4**. Molecular structures of the main organic compounds used in this study.

Supplementary Figures

Supplementary Figure **1**. The powder X-ray diffraction (XRD) patterns of HNP (a), HNR (b), and HNC (c).

Supplementary Figure **2**. The particle size distribution of HNP, HNR and HNC.

Supplementary Figure **3**. Surface zeta (ζ) potentials of HNP, HNR and HNC as a function of pH.

Supplementary Figure **4**. The X-ray photoelectron spectroscopy (XPS) of HNP.

Supplementary Figure **5**. The X-ray photoelectron spectroscopy (XPS) of HNR

Supplementary Figure **6**. The X-ray photoelectron spectroscopy (XPS) of HNC.

Supplementary Figure **7**. The Brunauer-Emmett-Teller (BET) method based N_2 adsorption-desorption isotherms for HNP (a), HNR (b), and HNC (c).

Supplementary Figure **8**. Transformation of DMP/DnBP on HNP, HNR, and HNC.

Supplementary Figure **9**. Mass spectra of DMP, DnBP and their hydrolytic products MMP, MnBP and PA (silanized) at positive ionization mode.

Supplementary Figure **10**. Hydrolysis kinetics of methyl benzoate (MB) on HNP (black), HNR (red), and HNC (blue).

Supplementary Figure **11**. The possible surface terminations of the hematite {001} facet before and after surface relaxation.

Supplementary Figure **12**. The possible surface terminations of the hematite {104} facet before and after surface relaxation.

Supplementary Figure **13**. The possible surface terminations of the hematite {012} facet before and after surface relaxation.

Supplementary Figure **14**. Bader charge analysis of the facet-exposed Fe.

Supplementary Figure **15**. *In-situ* DRIFTS measurements for surface Lewis-acid sites.

Supplementary Figure **16**. *In-situ* DRIFTS measurements by applying gaseous DMP.

Supplementary Figure **17**. Molecule geometries of *trans*-DMP, *cis*-DMP, *trans*-DnBP and *cis*-DnBP.

Supplementary Figure **18**. The adsorption configurations of DMP on {001}, {104} and {012} facets, as well as the corresponding calculated IR spectra.

Supplementary Figure **19**. The experimental IR spectra of DMP (a), and the theoretical IR spectra of *trans*-DMP (b,d) and *cis*-DMP (c, e) by Gaussian calculation (b, c) and VASP calculation (d, e).

Supplementary Figure **20**. The theoretical IR spectra of *trans-*/*cis*-DnBP before and after complexing with $Fe(OH)_3$ or $Fe_2O(OH)_4$ clusters.

Supplementary Figure **21**. The experimental IR spectra of pure DnBP (a), and the adsorbed DnBP on HNP (b), HNR (c), HNC (d), by *ex-situ* KBr wafer method.

Supplementary Figure **22.** The accumulative trend of the carbonyl stretching vibration ($\nu_{C=O}$) at 1565 - 1620 cm^{-1} on HNR (a, b) and HNC (c, d).

Supplementary Figure **23**. The nature bond orbital (NBO) charge distribution and Fe-O bond length of DMP at its monodentate-coordination mode (a) and bidentate-coordination mode (b).

Supplementary Figure **24**. The accumulative adsorption of DMP as well as its hydrolytic products (MMP and PA) on HNR (a) and HNC (b) under RH 76 % and at 35 ˚C.

Supplementary Figure **25**. The hydrolysis rate constants (k_{BET}) of DMP on HNR and HNC at different initial concentrations (2.0-20.0 $\mu mol \cdot g^{-1}$).

Supplementary Figure **26**. Catalytic performance of facet-controlled TiO_2 nanoparticles for hydrolyzing DMP.

Supplementary References

Supplementary Note 1. Characterizations about surface carbon residual. The isoelectric point of hematite can to some extent indicate its surface condition, because the isoelectric point of hematite could be sensitive to the adsorbed ions and ligands. Based on zeta (ζ) potential measurement, the isoelectric points for hematite nano-plate (HNP, majority {001} facet exposed), hematite nano-rhombohedra (HNR, single {104} facet exposed) and hematite nano-cube (HNC, single {012} facet exposed) nanoparticles were measured as 7.0 - 8.5 (Supplementary Fig. 3), close to the values reported in the literatures[1, 2]. For example, the isoelectric point of hematite {001} was reported as ~7.8[1], and the measured equivalent point of zero potential for hematite {001} and {012} were in the range of 8.35~8.65[2]. If the hematite surface was contaminated by organic carboxylic acid, the surface electrostatic potential would be significantly reduced. It was reported that the isoelectric point of hematite {001} would reduce to pH < 3 in the presence of 10 μM oleic acid[3]. It is obviously not the case for HNP in our study, as we used acetic acid for synthesis. Thus, the isoelectric points suggest that our synthesized hematite nanoparticles were unlikely contaminated by the organic ligands used in synthesis.

X-ray photoelectron spectroscopy (XPS) can provide extra evidence to examine whether the hematite surface was contaminated by organic. The XPS results of the three hematite (HNP, HNR and HNC) are shown in Supplementary Fig. 4-Fig. 6. Although all presented clear C *1s* peak, the fitted C compositions (C-C/C-H: 73-77%, C-N/C-O: 12-18%, C=O: 9-11%) do not match the chemical compositions of the organic ligands (CH_3COO^- for HNP, H_2NCONH_2 and $HCONH_2$ for HNR, CH_3COONH_4 for HNC). For

example, H_2NCONH_2 and $HCONH_2$ were used to synthesize HNR. While, there are no C-C and C-H bonds in the two ligands, and the synthesizing temperature of 160-180 °C is unlikely to cause carbonization of the organic ligands. In addition, since the organic ligands used for synthesis contain high amount of N element in the cases of HNR and HNC, the detection of N *1s* by XPS could also reflect whether the hematite surface was contaminated by the organic ligands or not. As shown in Supplementary Fig. 5 & Fig. 6, no N *1s* XPS signal was observed on HNR and HNC. Therefore, we can conclude the hematite surfaces are not contaminated by the organic ligands from synthesis.

To further verify this, we washed the hematite nanoparticles with ethanol and water for more than 10 times, then calcinated them at 400 °C for 2 h. However, the additional washing and calcination treatments still cannot remove the C *1s* signal. Referring to the literatures from different researchers, the residual organic carbon is commonly detectable in XPS analysis. For example, the synthesized hematite {001}, {012}, {110} nanoparticles also presented clear C *1s* XPS signal in the literatures[4-6], demonstrating carbon contamination on hematite surface is difficult to avoid. Since the C compositions on the three hematite are very similar, the carbon contamination might stem from extra sources after synthesis. However, the discussion for the extra source of the carbon contamination is beyond the scope of this study, further investigation should be conducted.

Based on the above discussion, the collective XPS and isoelectric points results show that the three synthesized hematite are pure, with slight adventitious carbon contamination. However, the composition of C=O species is low (9-11%). Since only

the C=O species can compete for the Lewis-acid sites, the C-C/C-H and C-N/C-O species have poor coordination capability, the slight carbon contamination should have little influence on the hydrolysis reaction. It would not challenge the main conclusion of current study.

Supplementary Note 2. Calculations of the single Fe site density (D_{si}) and the "probability bidentate Fe-Fe site density" ($D_{p\text{-}bi}$). We suppose one exposed Fe site can coordinate with one ester group. We define D_{si} as the average Fe site number (N) on the unit surface area (A), $D_{si} = N/A$. Therefore, the $D_{si\text{-}\{001\}}$ and $D_{si\text{-}\{012\}}$ were calculated to be 4.51 and 7.25 sites·nm^{-2}, respectively. The {104}-layer 1 termination exposed with two layers of Fe (marked in yellow for the outmost Fe, and cyan for the subsurface Fe). The outmost Fe layer is just ~1.6 Å above the subsurface Fe layer. Both the $Fe_{outmost}$ and the $Fe_{subsurface}$ are four-fold coordinated, and should be accessible for monodentate coordination. Thus, the $D_{si\text{-}\{104\}}$ is calculated to be 10.62 sites·nm^{-2}.

The bidentate coordination is more complicated. One *cis*-DMP/DnBP compound needs to coordinate with two neighboring Fe sites. For the {001} facet, the neighboring Fe-Fe distance (5.063 Å) on its layer 1 termination is too wide to coordinate with the two ester groups of DMP/DnBP. For the {012} facet, there are 8 Fe atoms on unit surface area (1.0122 nm × 1.0905 nm), and each Fe atom is adjacent to 4 neighboring Fe atoms (A-B, A-C, A-D, and A-E) with the distance of 0.35 - 0.39 nm. Two Fe atoms comprise one bidentate coordination site. Therefore, the $D_{p\text{-}bi\text{-}\{012\}}$ is calculated to be 14.50 sites·nm^{-2}. For the {104}-facet, the outmost Fe atoms is more accessible for

coordination. While, the subsurface Fe atoms process stronger Lewis acidity. The calculated adsorption configurations of DMP on the {104} facet confirmed that one of the coordination sites always locates at the bridging position of one outmost Fe and one subsurface Fe (Supplementary Fig. 18b-c). Due to steric hinderance, it is difficult to form bidentate coordination with two subsurface Fe atoms. According to this model, the {104} facet (layer 1 termination) could be divided into 6 planar hexagonal regions, each region provides two bidentate-coordination sites. Therefore, the $D_{p\text{-}bi\text{-}\{104\}}$ is calculated to be 7.97 sites·nm^{-2}.

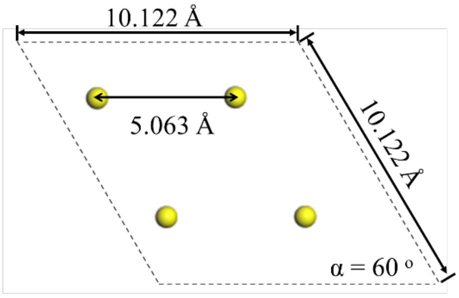

a.{001}-layer 1

$$D_{si} = \frac{4}{10.122 \times 10.122 \times \sin(60^o)} = 4.51 \text{ site} \cdot \text{nm}^{-2}$$

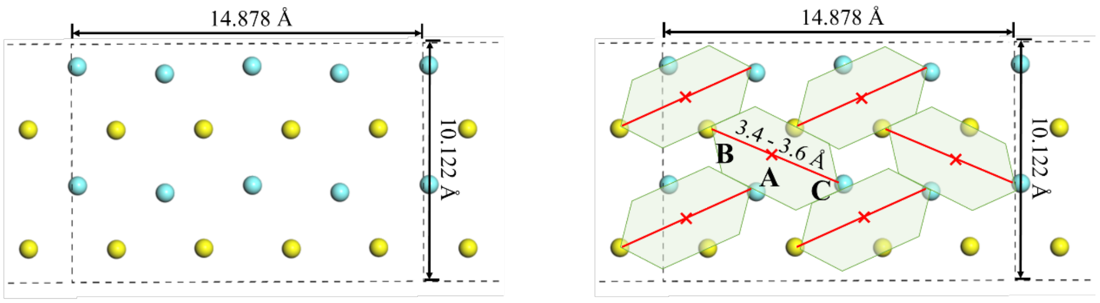

b.{104}-layer 1

$$D_{si} = \frac{16}{14.878 \times 10.122} = 10.62 \text{ site} \cdot \text{nm}^{-2} \quad D_{p-di} = \frac{6 \times 2}{14.878 \times 10.122} = 7.97 \text{ site} \cdot \text{nm}^{-2}$$

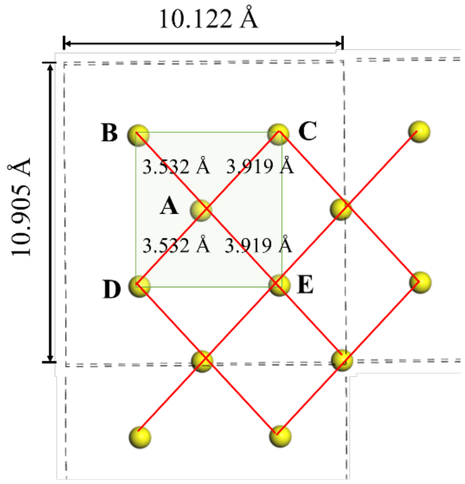

c.{102}-layer 1

$$D_{si} = \frac{8}{10.122 \times 10.905} = 7.25 \text{ site} \cdot \text{nm}^{-2}$$

$$D_{p-di} = \frac{8 \times 4/2}{10.122 \times 10.905} = 14.50 \text{ site} \cdot \text{nm}^{-2}$$

Supplementary Method 1. Chemicals and reagents. Dimethyl phthalate (DMP, 99%) and di-n-butyl phthalate (DnBP, 97%) were purchased from TCI (Tokyo, Japan); monomethyl phthalate (MMP, 97%), mono-n-butyl phthalate (MnBP, 98%), phthalic acid (PA, 99.5%), and methyl benzoate (MB, 99.5%) were from Aladdin Biochemical Technology (Shanghai, China). The TiO2 nanopowder (CAS 13463-67-7), which is amorphous, was purchased from Shanghai Lingfeng Chemical Research Co. Ltd. (Shanghai, China). Other inorganic salts and reagents are of analytical or higher grade.

Supplementary Method 2. Methods for synthesizing HNP/HNR/HNC. Briefly, for HNP, $FeCl_3 \cdot 6H_2O$ (4 mmol) and CH_3COONa (40 mmol) were dissolved in 40.0 mL ethanol with a trace addition of 2.8 mL deionized water (18.2 MΩ·cm), then the mixture was sealed in a Teflon-lined autoclave and maintained at 180 °C for 12 h. For HNR,

$Fe(NO_3)_3·9H_2O$ (7 mmol), urea (H_2NCONH_2, 35 mmol) and formamide ($HCONH_2$, 70 mmol) were dissolved in 70.0 mL deionized water, and the mixture was maintained at 160 °C for 24 h inside a Teflon-lined autoclave. HNC was synthesized by mixing $FeCl_3·6H_2O$ (1.8 mmol) and CH_3COONH_4 (90 mmol) in 24.0 mL deionized water, and the solution pH was adjusted to 11 by adding $NH_3·H_2O$ dropwise with continuous stirring for 30 min, then, the suspension was maintained at 160 °C for 24 h in a Teflon-lined autoclave. After cooling down to room temperature, the synthesized HNP, HNR and HNC powders were washed by water and ethanol alternately for at least 5 times to remove residual ligands or ions. Then, the hematite nanoparticles were freeze-dried for further use.

Supplementary Method 3. Characterization methods. The powder X-ray diffraction (XRD) patterns of the HNP, HNR, and HNC were scanned by a D8 Advance X-ray diffractometer (Bruker, Germany) with a Cu Kα radiation source performed at 40 kV and 40 mA (2θ from 10° to 80°). The specific surface areas (SSA) of the synthesized hematite were measured by a surface area analyzer (ASAP 2460, Micromeritics, USA) utilizing the N_2 adsorption−desorption Brunauer−Emmett−Teller (BET) method. The morphologies of particles were observed by scanning electron microscopy (SEM, FEI-Quanta FEG 250, Thermo Fisher Scientific., USA) under 15 kV. The transmission electron microscopy (TEM) images were obtained by a H7650 microscope (Hitachi, Japan) at the acceleration voltage of 200 kV with a selected area electron diffraction (SAED) scan. The particle size distribution of HNP, HNR and HNC were measured by

a laser particle size analyzer (ZEN 3500 zetasizer), at 100 mg/L and pH = 4.0 without pH buffer. The same instrument was used for measuring the surface zeta (ζ) potential of HNP, HNR and HNC at 100 mg/L with respect to varied pH. The pH was adjusted by HCl or NaOH. The surface elemental composition (Fe *2p*, O *1s*, C *1s*, N *1s*) was characterized by X-ray photoelectron spectroscopy (XPS) (PHI 5000, VersaProbe-II, Ulvac-Phi Inc., Japan). The contributions of different oxygen species for simulating O *1s* were fitted according to Schöttner et al.[7] And the C *1s* compsition was fitted according to the NIST X-ray photoelectron spectroscopy database (https://srdata.nist.gov/xps/Default.aspx)

Supplementary Method 4. High performance liquid chromatography (HPLC) analytical methods. The residual DMP, DnBP, and the products were quantified by HPLC equipped with an ultraviolet detector (Waters 2998 Photodiode Array Detector, USA). An Eclipse XDB-C18 column (5 μm, 4.6 × 150 mm, Agilent) was used for separation. The mobile phase was composed by acetonitrile and water (containing 0.01% trifluoroacetic acid). The flow rate was set at 1.2 $mL \cdot min^{-1}$. Separation was achieved by running the gradient elution program (acetonitrile%, vol.%)[8]: (1) for DMP and its hydrolytic products, running 15% acetonitrile for 5 min, increasing to 40% acetonitrile in the following 10 min, then adjusting back to 15% acetonitrile in the next 5 min and maintaining at 15% for another 5 min; (2) for DnBP and its hydrolytic products, running 15% acetonitrile for 5 min, increasing to 40% acetonitrile in the following 10 min and maintaining at 40% for another 10 min, afterward, increasing the acetonitrile to 85% in

10 min and maintaining at 85% for another 10 min, finally decreasing to 15% acetonitrile in 5 min and lasting for 5 min. DMP, DnBP and their products were detected at 254 nm. The analytical condition for MB was different. It was eluted by methanol and water (60:40, v:v) at a flow rate of 1.0 mL·min^{-1}, and was detected at 230 nm.

Supplementary Method 5. Gas chromatography mass spectrometer (GC-MS) analytical method. The hydrolytic products of DMP and DnBP were identified by GC-MS (Thermo scientific, US). The water sample was extracted by dichloromethane, and dehydrated by anhydrous sodium sulfate, then derivatized by N,O-bis-(trimethylsilyl)-trifluoroacetamide (BSTFA)/trimethylchlorosilane (TMCS) (99:1) in a ratio of 1 : 1 (v : v, 0.5 mL : 0.5mL) under 60 ˚C for 30 min. The heating program of GC-MS was starting from 60 ˚C to 280 ˚C with a rate of 10 ˚C ·min^{-1}, then holding for 5 min.

Supplementary Table 1. Surface energies (γ) for individual facet termination.

Facet termination	E_{bulk} eV)	n	$E_{\mathrm{slab}}^{\mathrm{unrelax}}$ (eV)	$E_{\mathrm{slab}}^{\mathrm{relax}}$ (eV)	A (Å^2)	γ ^{a} (J·m^{-2})
{001}-Layer 1, Fe_3-O_3-Fe_6-R			-515.17	-518.90		1.86
{001}-Layer 2, O_2-Fe_6-Fe_6-R	-203.46 ($Fe_{12}O_{18}$)	2.67	-497.34	-499.50	88.73	3.69
{001}-Layer 3, Fe_3-Fe_3-O_4-R			-495.91	-298.13		3.81
{014}-Layer 1, O_3-Fe_4-Fe_4-O_4-O_4-R			-767.80	-772.55		1.94
{014}-Layer 2, O_3-Fe_4-Fe_2-O_4-O_4-R			-727.21	-734.82		3.80
{014}-Layer 3, O_2-Fe_4-O_4-O_4-Fe_6-R	-203.46 ($Fe_{12}O_{18}$)	4	-761.14	-763.57	150.60	2.54
{014}-Layer 4, O-O_2-O_3-Fe_6-Fe_6-R			-727.53	-732.18		4.10
{014}-Layer 5, O_2-O_3-Fe_5-Fe_6-O_3-R			771.51	772.24		2.12
{012}-Layer 1, O_3-Fe_5-O_4-Fe_6-O_4-R			-781.18	-784.08		1.95
{012}-Layer 2, O_3-Fe_3-O_4-Fe_4-O_4-R			-758.92	-759.20		3.94
{012}-Layer 3, O_2-Fe_5-O_3-O_4-Fe_6-R	-203.46 ($Fe_{12}O_{18}$)	4	-768.99	-769.86	110.38	3.13
{012}-Layer 4, O_3Fe_3-O_4-Fe_6-O_4-R			-770.49	-773.70		2.68
{012}-Layer 5, O-O_3-Fe_6-O_4-Fe_6-R			-751.03	-752.30		4.37

a. The surface energy (γ) comprised by the cleavage energy (E_{cut}) and the reconstruction energy (E_{relax})[9]. Namely, $\gamma = E_{cut} + E_{relax} = \frac{1}{2A}(E_{slab}^{unrelax} - nE_{bulk}) + \frac{1}{A}(E_{slab}^{relax} - E_{slab}^{unrelax})$, where $E_{slab}^{unrelax}$ is the unrelaxed energy of the fixed slab; E_{bulk} is the energy of the fully

optimized bulk structure ($Fe_{12}O_{18}$); *2A* denotes to the area of top and bottom surfaces after the cleavage; n is the total number of $Fe_{12}O_{18}$ units contained in the slab model; E_{slab}^{relax} is the relaxed energy of the optimized slab with only the top surface relaxed.

Supplementary Table 2. The BET-SSA normalized hydrolysis rate constants of DMP and DnBP on HNP, HNR and HNC, in proportional to the surface site density.

	Facet	k_{BET} (10^{-3} g·day^{-1}·m^{-2})					Surface site density (site·nm^{-2})	
		DMP		DnBP		MB	Single Fe site (D_{si})	Bidentate Fe-Fe site ($D_{p\text{-}bi}$)
		RH 76%	Water saturate	RH 76%	Water saturate	RH 76%		
HNP	84.41% {001}<br>15.59% {012}	1.91	0.09	0.51	0.07	79.04	4.94 average	NA
HNR	100% {104}	20.38	0.99	11.70	0.35	165.16	10.62 {104}	7.97 {104}
HNC	100% {012}	41.76	1.58	25.51	0.59	115.55	7.25 {012}	14.50 {012}
Ratio		$k_{BET\text{-}\{012\}}$: $k_{BET\text{-}\{104\}}$<br>2.05 : 1.00 (76%)<br>1.59 : 1.00 (water saturate)		$k_{BET\text{-}\{012\}}$: $k_{BET\text{-}\{104\}}$<br>2.18 : 1.00 (76%)<br>1.69 : 1.00 (water saturate)		$k_{BET\text{-}\{012\}}$: $k_{BET\text{-}\{104\}}$: $k_{BET\text{-}\{001\}}$<br>1.46 : 2.09 : 1.00	$D_{si\text{-}\{012\}}$: $D_{si\text{-}\{104\}}$: $D_{si\text{-}\{001\}}$<br>1.47 : 2.14 : 1.00	$D_{p\text{-}bi\text{-}\{012\}}$: $D_{p\text{-}bi\text{-}\{104\}}$<br>1.82 : 1.00

Supplementary Table 3. The calculated turnover number (TON) and turnover frequency (TOF) values of the HNP, HNR and HNC for the hydrolytic degradation of DMP, DnBP and MB under RH 76%.

	k_{BET} (10^{-3} g·day^{-1}·m^{-2})			SSA	D_{si}	$D_{p\text{-}bi}$	TON (10^{-3}) [a]			TOF (10^{-3} day^{-1}) [b]		
	DMP	DnBP	MB	(m^2·g^{-1})	(site·m^{-2})	(site·m^{-2})	DMP	DnBP	MB	DMP	DnBP	MB
HNP	1.91	0.51	79.04	24.9	4.94E18	NA	0.46 [c]	0.12 [c]	8.41 [c]	0.47 [c]	0.12 [c]	19.3 [c]
HNR	20.38	11.70	165.06	11.4	1.06E19	7.97E18	2.75 [d]	1.65 [d]	8.42 [c]	3.08 [d]	1.77 [d]	18.7 [c]
HNC	41.76	25.51	115.55	18.1	7.25E18	1.45E19	2.43 [d]	1.70 [d]	8.04 [c]	3.47 [d]	2.12 [d]	19.1 [c]

Note: *a*. TON = $\frac{C_0 \cdot (1\text{-}exp(\text{-}k_{BET} \cdot SSA \cdot t)) \cdot N_A}{D_{si}\ or\ D_{p\text{-}di} \cdot SSA}$; The TON was calculated after reacting for 1 d, t = 1 d; C_0 is the initial concentration of 2 μmol·g^{-1}; N_A = 6.02 × 10^{23}.

b. TOF = $\frac{C_0 \cdot k_{BET} \cdot N_A}{D_{si}\ or\ D_{p\text{-}di}}$; The TOF of the initial stage is calculated.

c. These TOF and TON values were calculated based on the D_{si}.

d. The TOF and TON of DMP/DnBP on HNR and HNC were calculated based on the $D_{p\text{-}bi}$.

Supplementary Table 4. Molecular structures of the main organic compounds used in this study.

Chemical structure	Molecular formula	CAS	Compound	Vapor pressure (mm Hg, 298 K)[10]
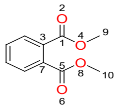	$C_{10}H_{10}O_4$	131-11-3	Dimethyl phthalate (DMP)	2.0×10^{-3} (liquid)
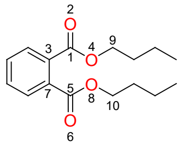	$C_{16}H_{22}O_4$	84-74-2	Di-n-butyl phthalate (DnBP)	2.7×10^{-5} (liquid)
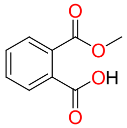	$C_9H_8O_4$	4376-18-5	Monomethyl phthalate (MMP)	
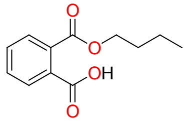	$C_{12}H_{14}O_4$	131-70-4	Mono-n-butyl phthalate (MnBP)	
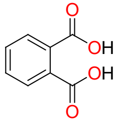	$C_8H_6O_4$	88-99-3	Phthalic acid (PA)	
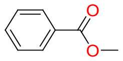	$C_8H_8O_2$	93-58-3	Methyl benzoate (MB)	

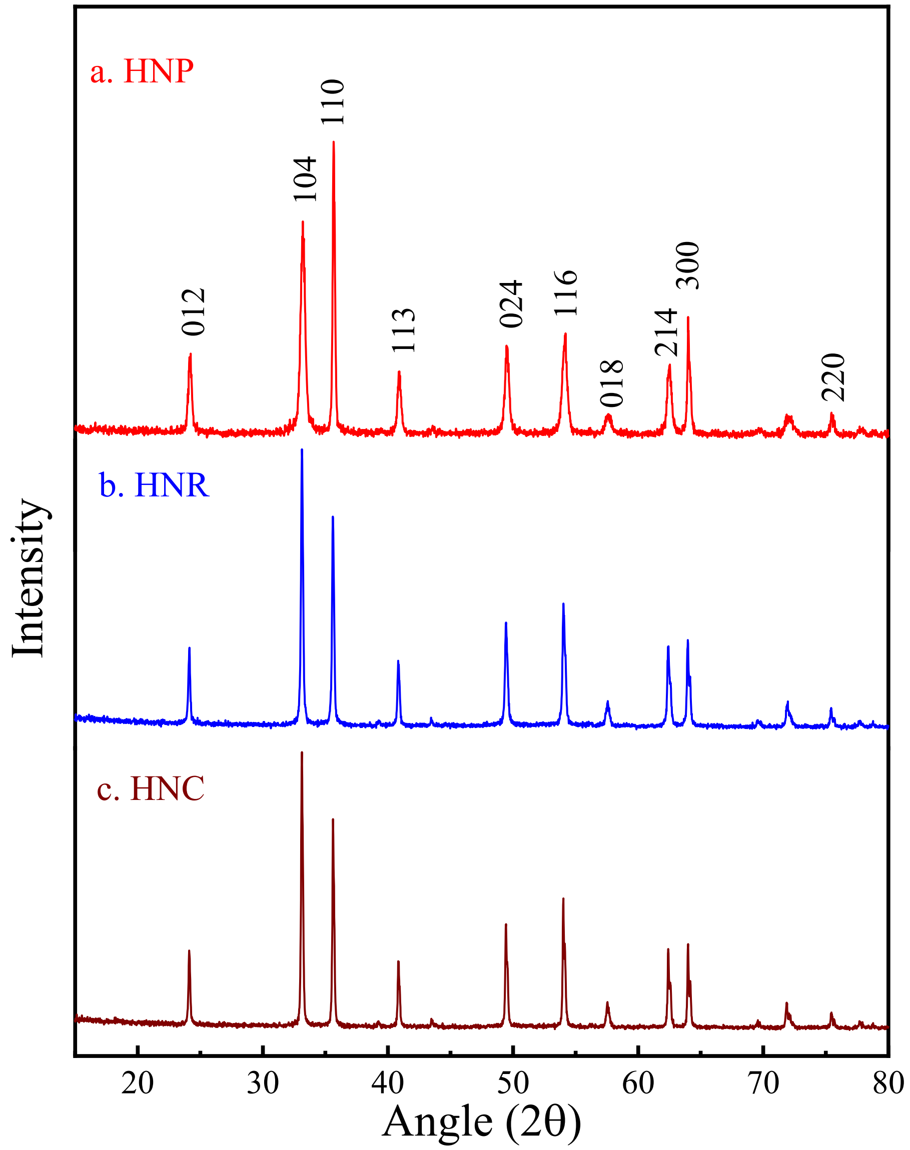

Supplementary Figure 1. The powder X-ray diffraction (XRD) patterns of HNP (**a**), HNR (**b**), and HNC (**c**).

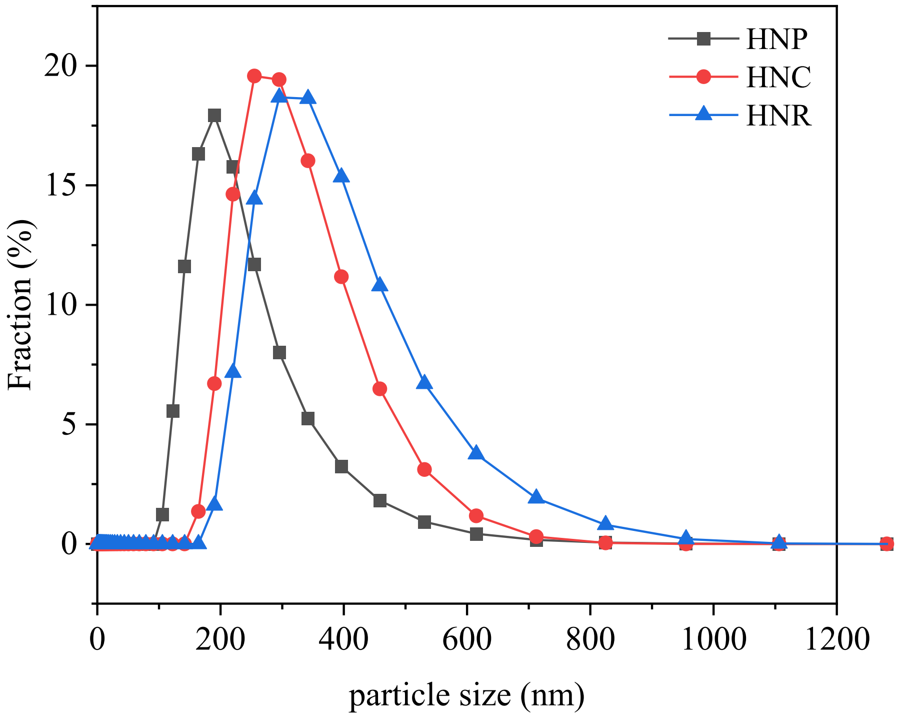

Supplementary Figure 2. The particle size distribution of HNP, HNR and HNC.

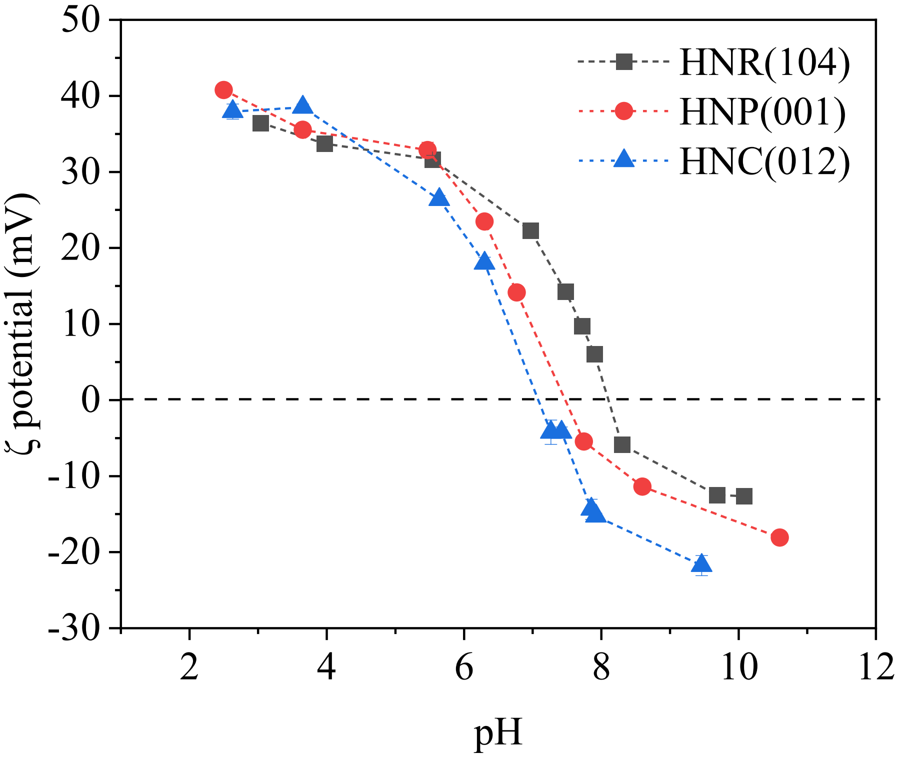

Supplementary Figure 3. Surface zeta (ζ) potentials of HNP, HNR and HNC as a function of pH. The isoelectric point is indicated by the point of $\zeta = 0$. The error bars are expressed as the mean value with standard deviations of 5 measurements.

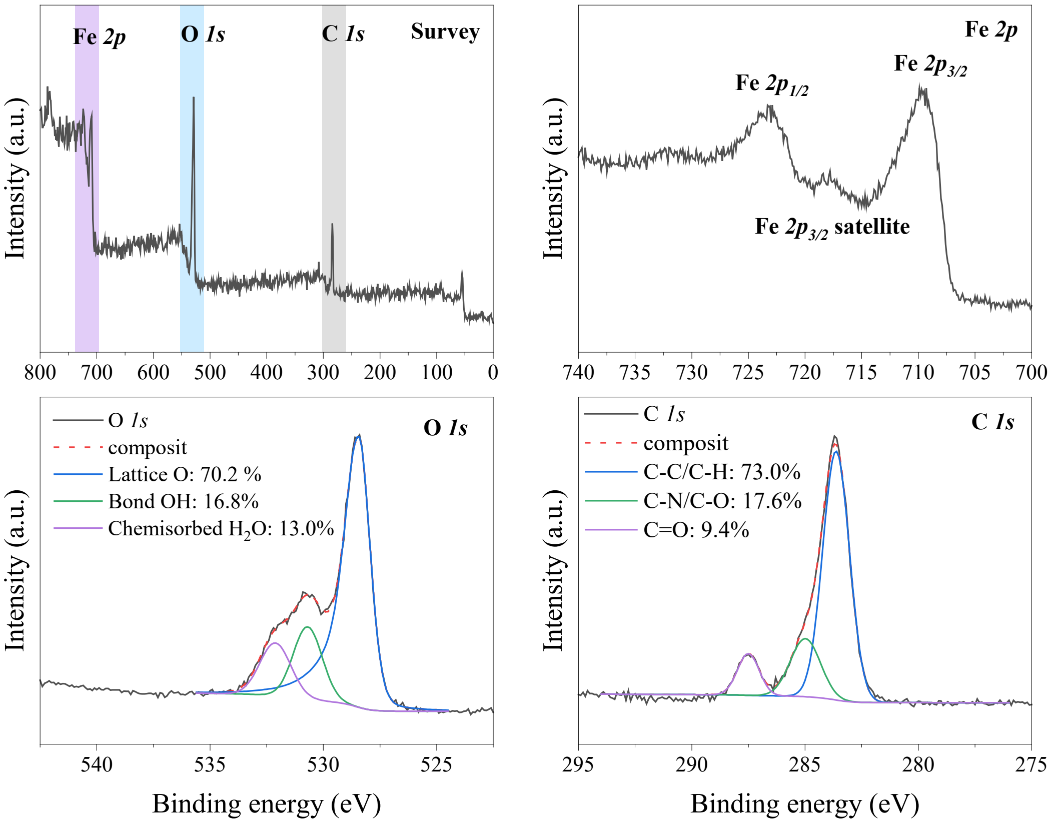

Supplementary Figure 4. The X-ray photoelectron spectroscopy (XPS) of HNP, including the survey spectra, high resolution spectra of Fe *2p*, O *1s*, and C *1s* XPS. The contributions of different oxygen species for simulating O *1s* were fitted according to Schöttner et al.[7] The composition of C species for C *1s* refers to the NIST X-ray photoelectron spectroscopy database (https://srdata.nist.gov/xps/Default.aspx).

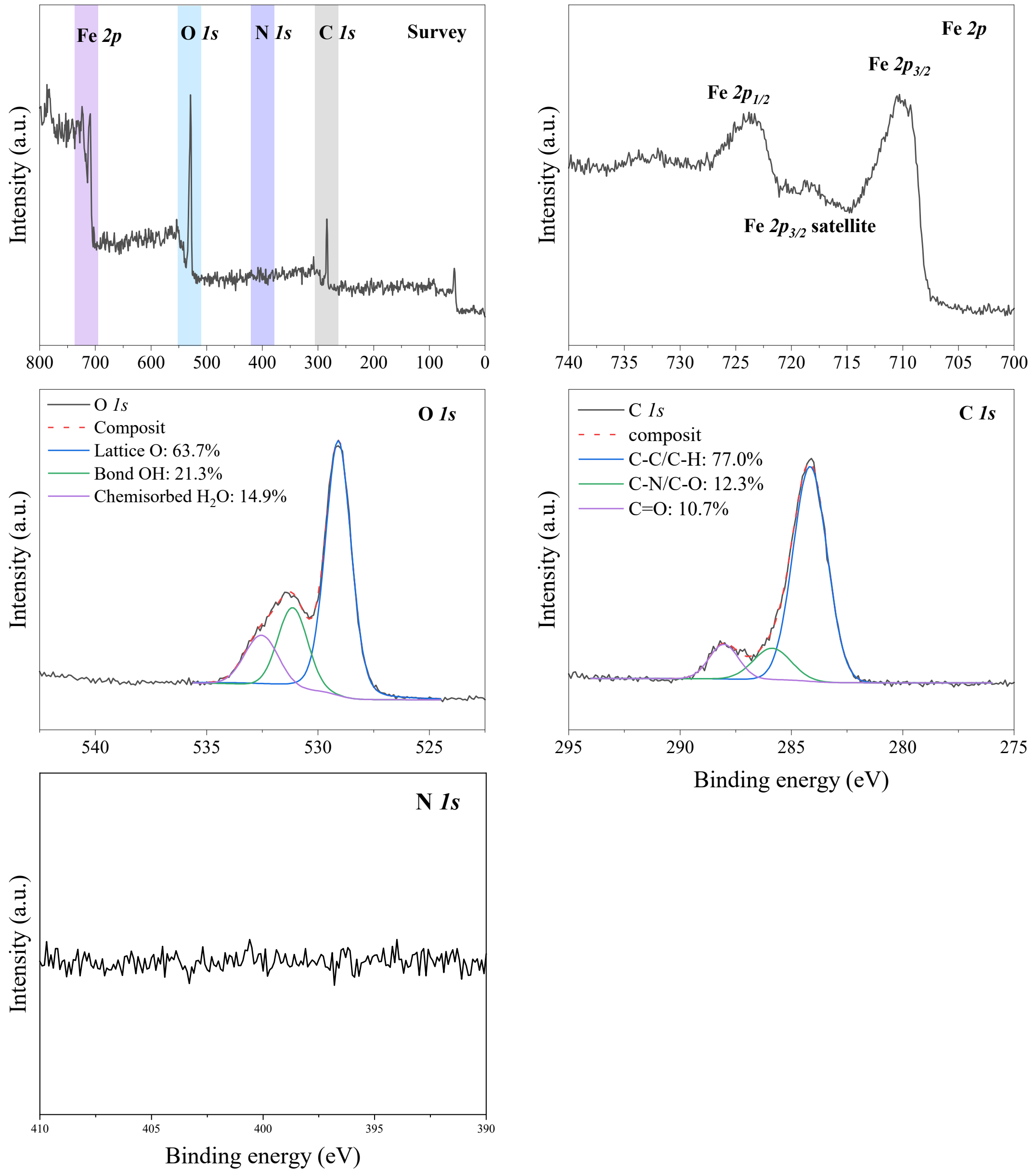

Supplementary Figure 5. The XPS of HNR, including the survey spectra, high resolution spectra of Fe *2p*, O *1s*, C *1s* and N *1s* XPS. The contributions of different oxygen species for simulating O *1s* were fitted according to Schöttner et al.[7] The composition of C species for C *1s* refers to the NIST X-ray photoelectron spectroscopy database (https://srdata.nist.gov/xps/Default.aspx).

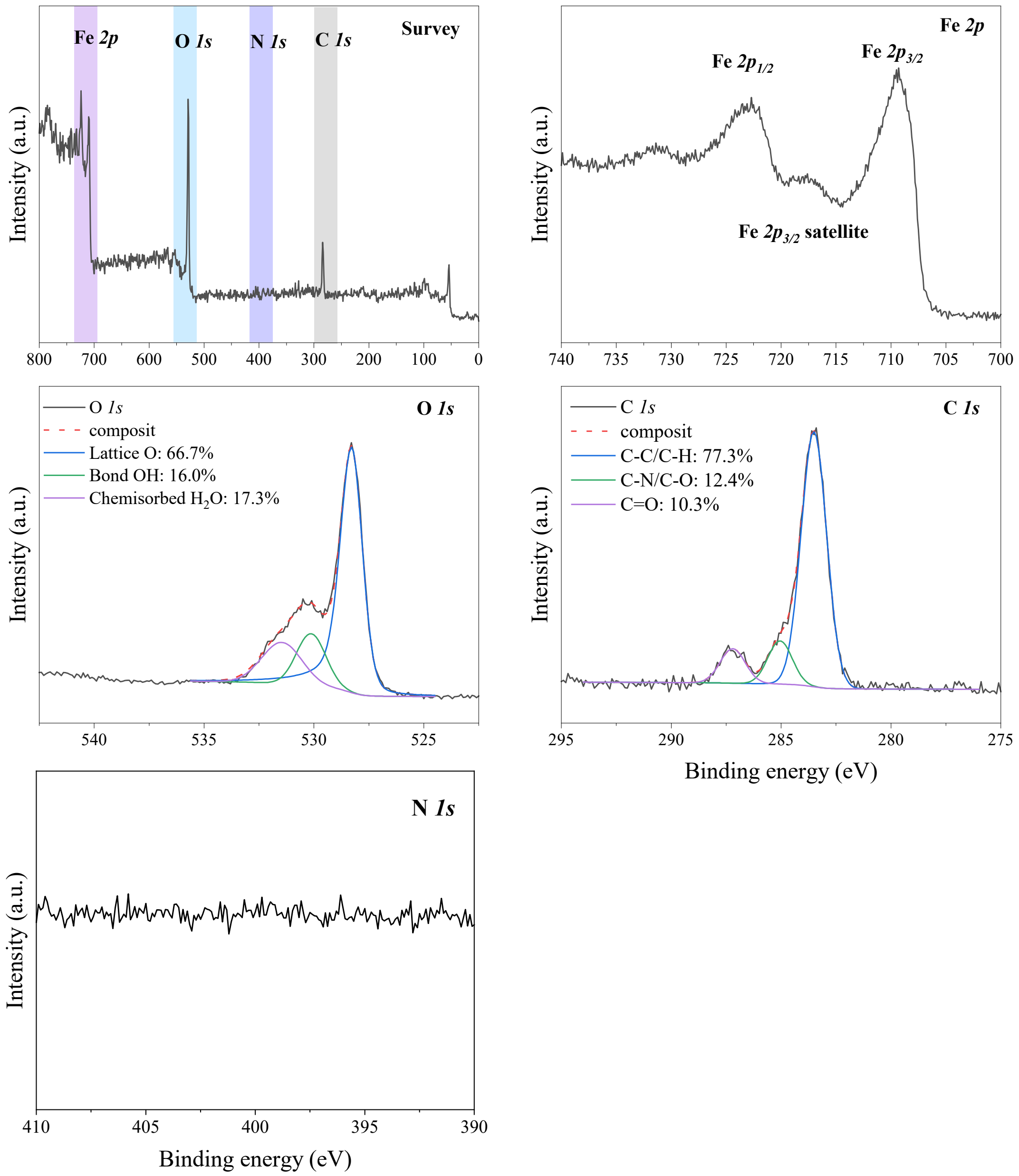

Supplementary Figure 6. The XPS of HNC, including the survey spectra, high resolution spectra of Fe *2p*, O *1s*, C *1s* and N *1s* XPS. The contributions of different oxygen species for simulating O *1s* were fitted according to Schöttner et al.[7] The composition of C species for C *1s* refers to the NIST X-ray photoelectron spectroscopy database (https://srdata.nist.gov/xps/Default.aspx).

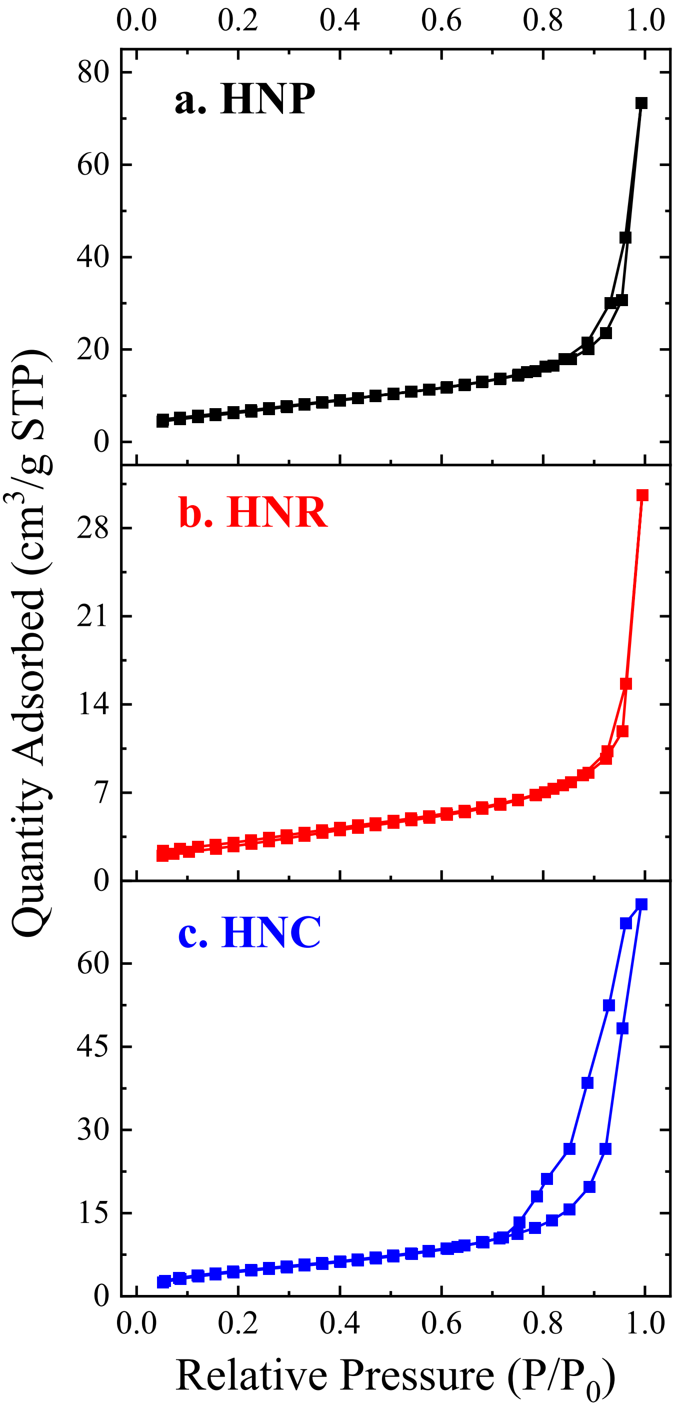

Supplementary Figure 7. The Brunauer-Emmett-Teller (BET) method based N_2 adsorption-desorption isotherms for HNP (**a**), HNR (**b**), and HNC (**c**).

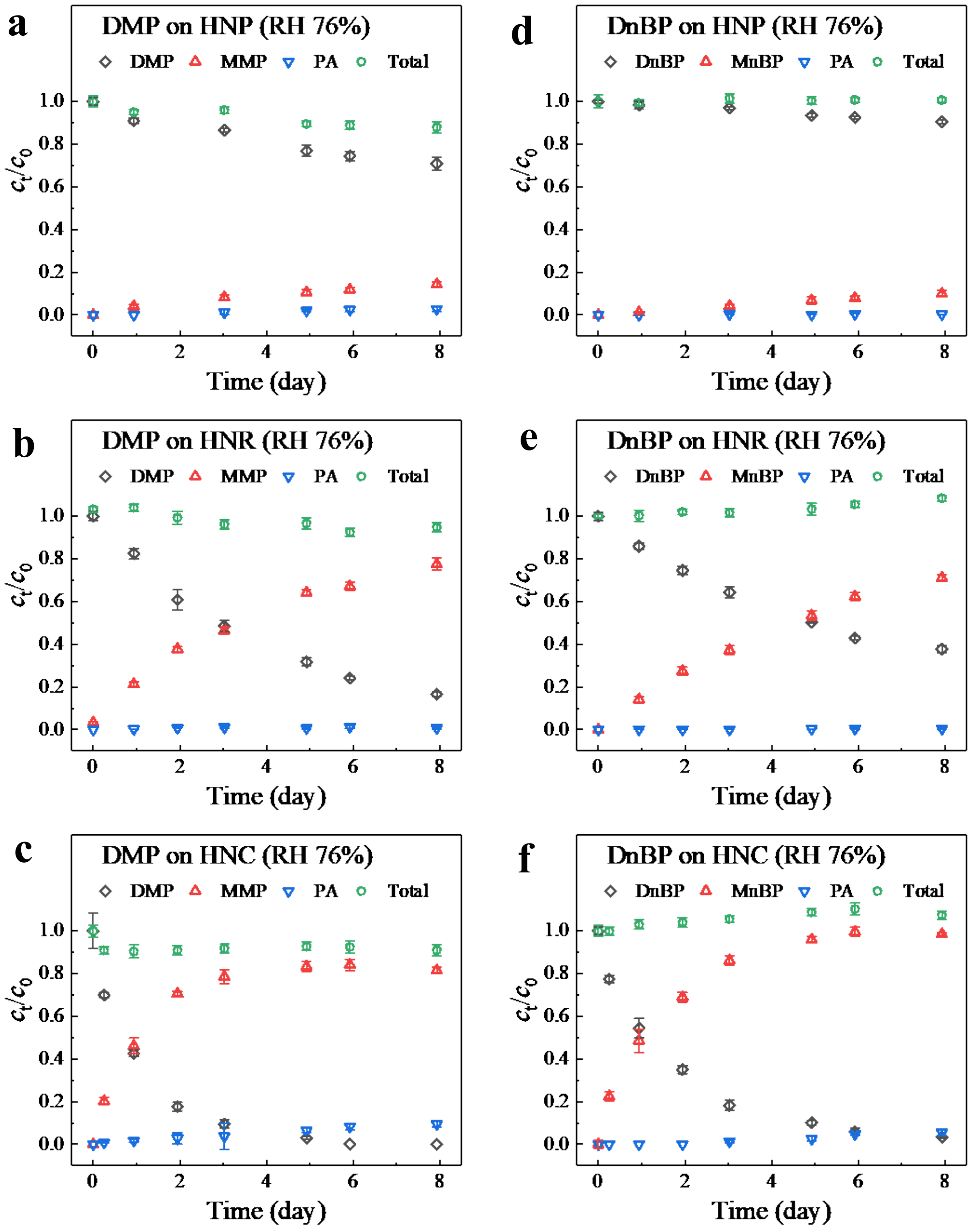

Supplementary Figure 8. **Transformation of DMP/DnBP on HNP, HNR, and HNC.** Degradation of DMP (**a, b, c**) and DnBP (**d, e, f**) on HNP (**a, d**), HNR (**b, e**), HNC (**c, f**) as a function of reaction time; The yields of the primary product of monomethyl phthalate (MMP) or mono-*n*-butyl phthalate (MnBP), and the secondary product of phthalic acid (PA) as a function of reaction time. The mass balance (Total) were presented. The error bars are expressed as the mean value with standard deviation of 2 experimental replicates. Source data are provided as a Source Data file.

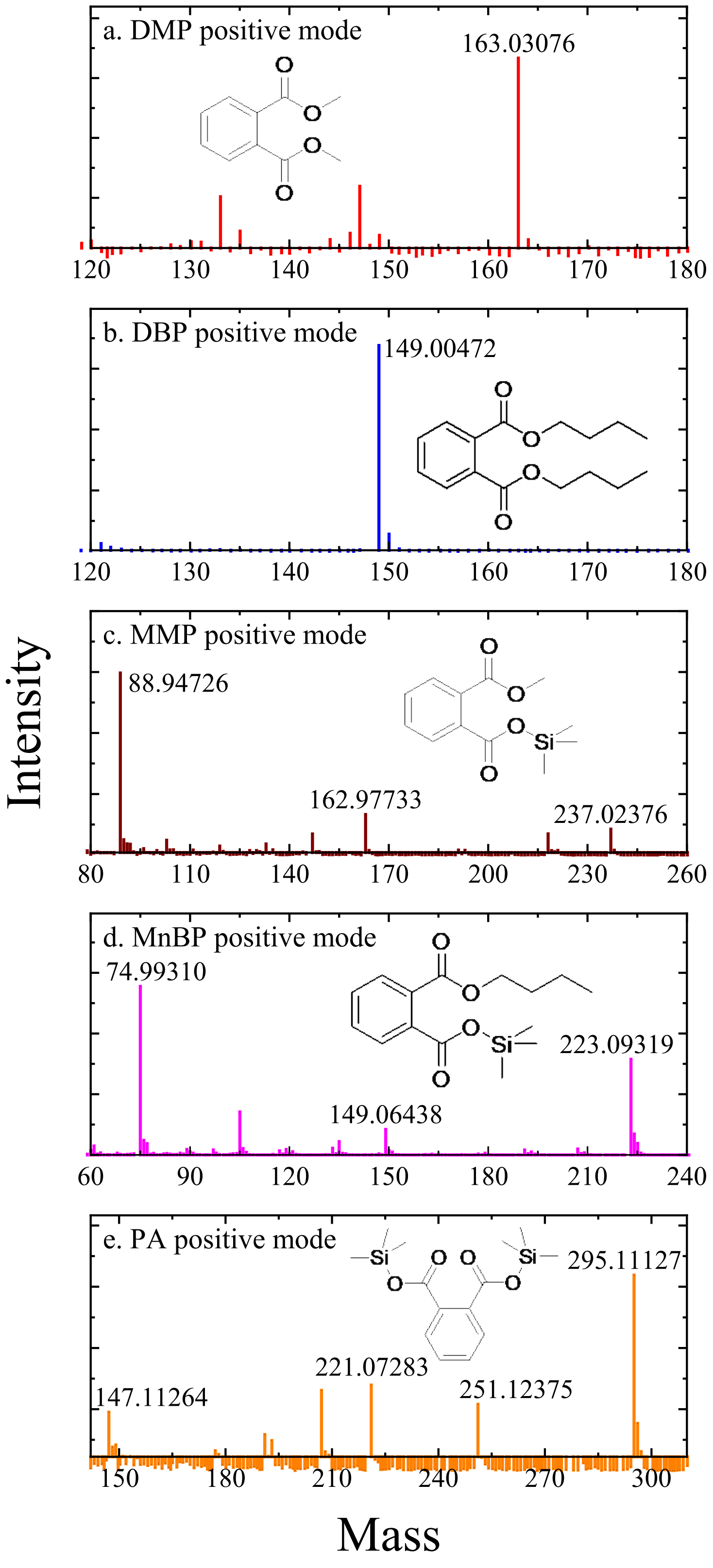

Supplementary Figure 9. Mass spectra of DMP, DnBP and their hydrolytic products MMP, MnBP and PA (silanized) at positive ionization mode.

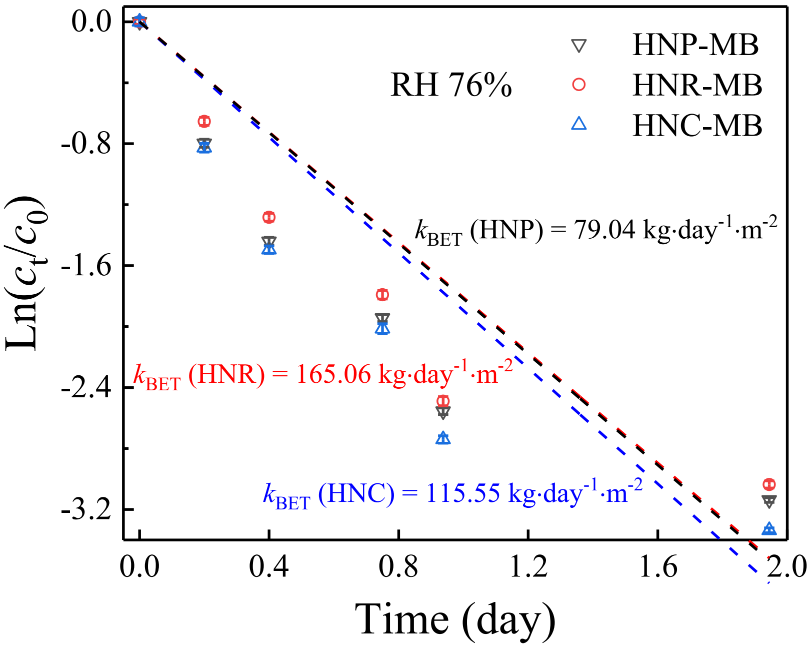

Supplementary Figure 10. Hydrolysis kinetics of methyl benzoate (MB) on HNP (black), HNR (red), and HNC (blue). The kinetics were fitted by the first-order kinetic model. The error bars are expressed as the mean value with standard deviations of 2 experimental replicates. Source data are provided as a Source Data file.

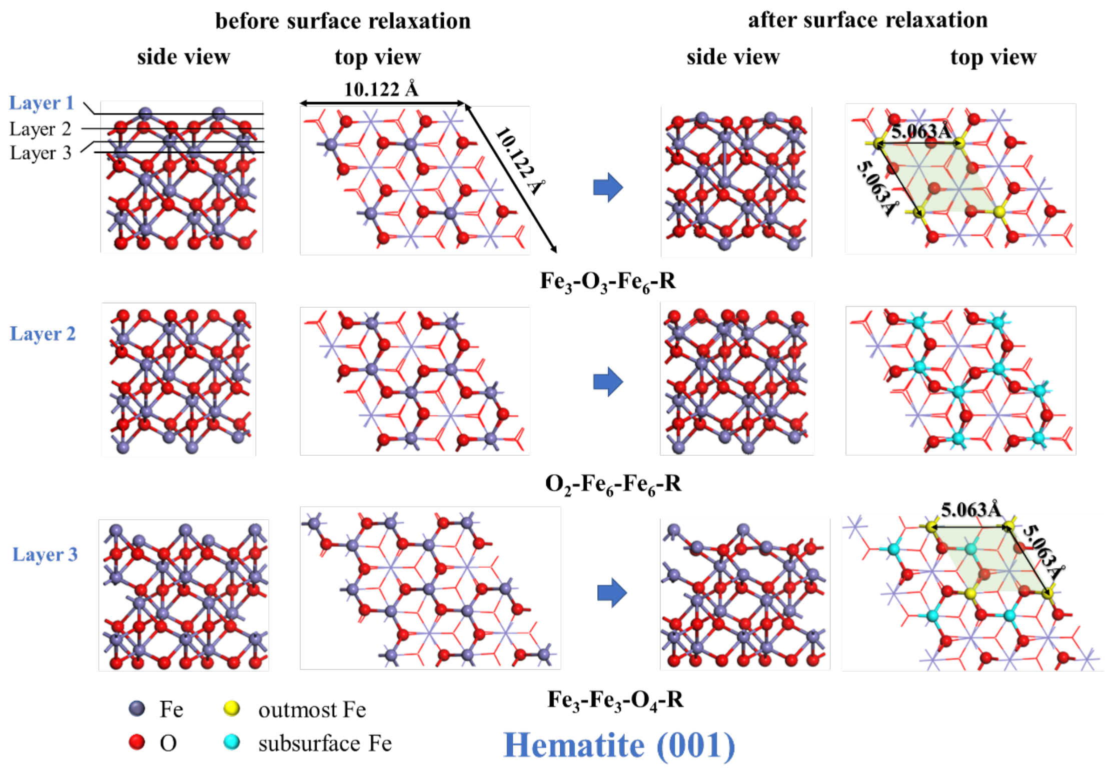

Supplementary Figure 11. The possible surface terminations of the hematite {001} facet before and after surface relaxation.

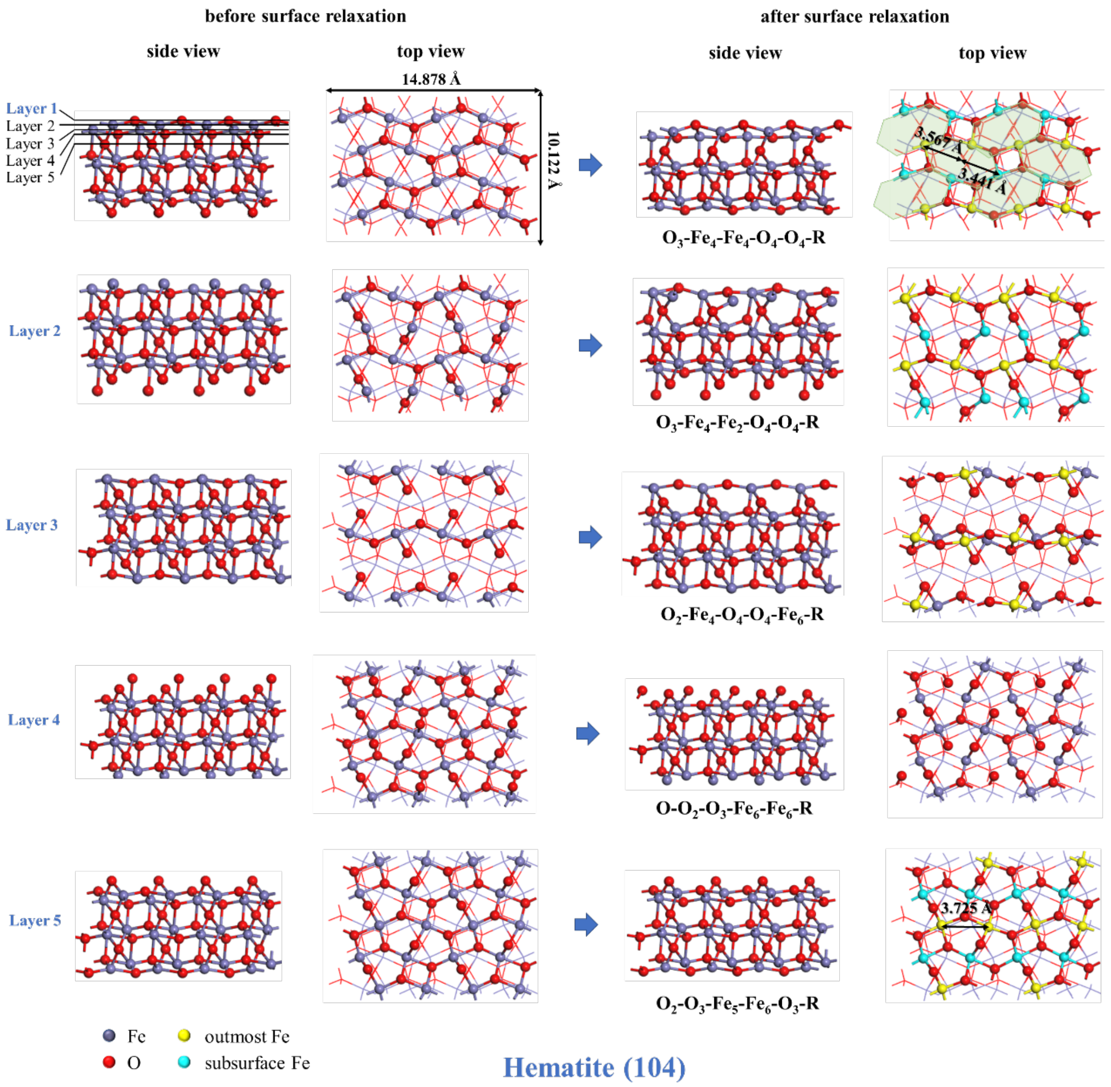

Supplementary Figure 12. The possible surface terminations of the hematite {104} facet before and after surface relaxation.

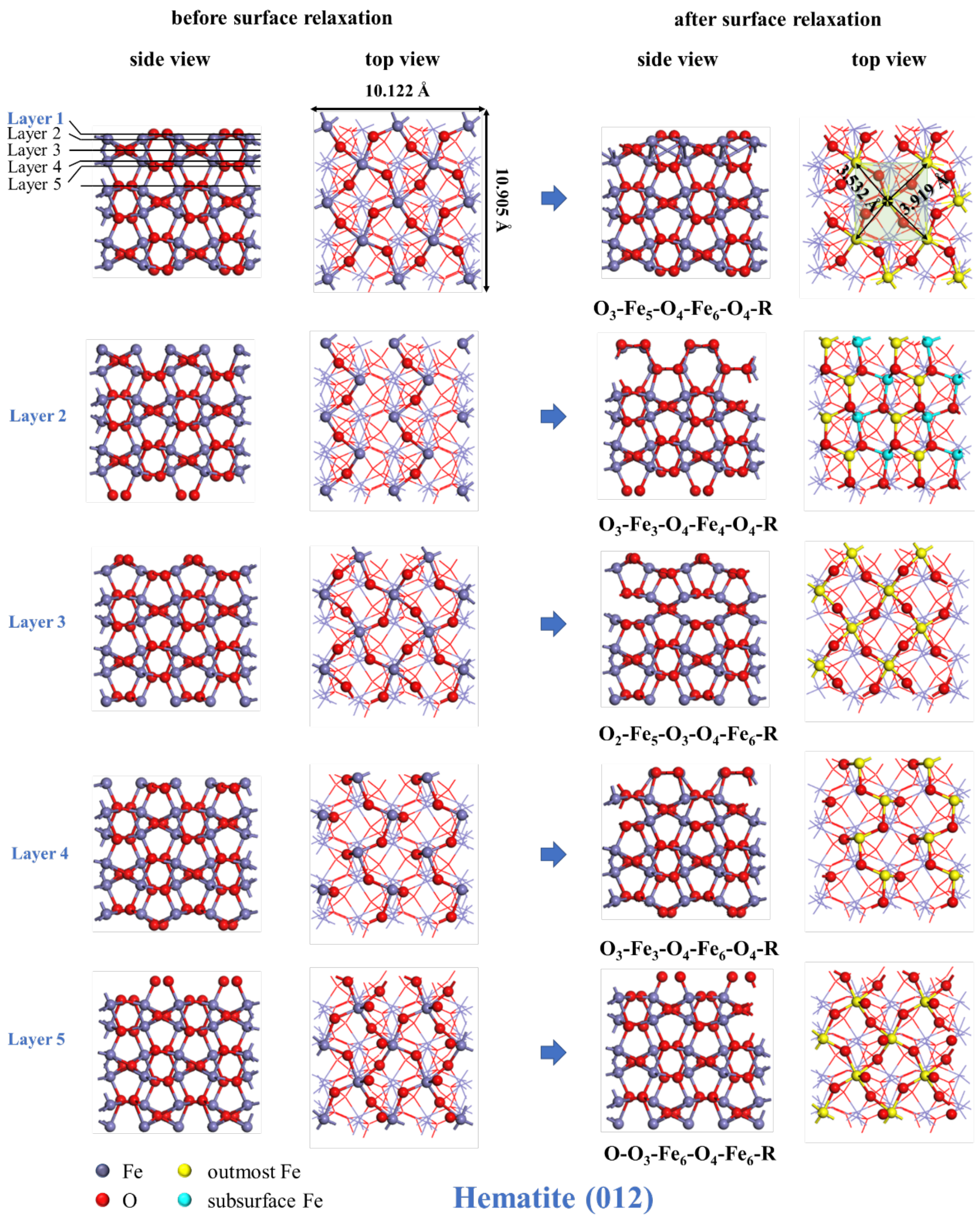

Supplementary Figure 13. The possible surface terminations of the hematite {012} facet before and after surface relaxation.

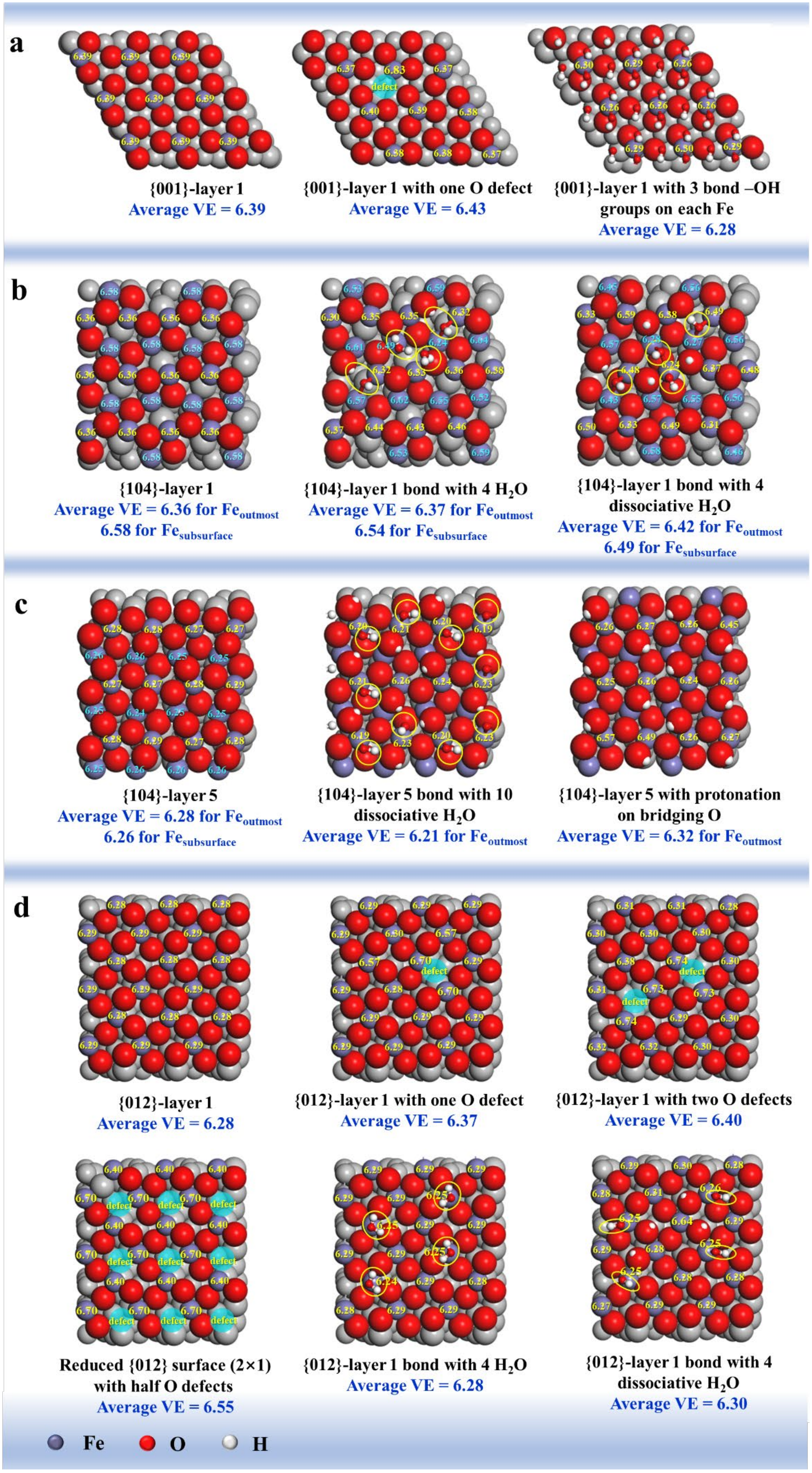
a
{001}-layer 1
Average VE = 6.39
{001}-layer 1 with one O defect
Average VE = 6.43
{001}-layer 1 with 3 bond –OH groups on each Fe
Average VE = 6.28
b
{104}-layer 1
Average VE = 6.36 for Fe$_{outmost}$
6.58 for Fe$_{subsurface}$
{104}-layer 1 bond with 4 H_2O
Average VE = 6.37 for Fe$_{outmost}$
6.54 for Fe$_{subsurface}$
{104}-layer 1 bond with 4 dissociative H_2O
Average VE = 6.42 for Fe$_{outmost}$
6.49 for Fe$_{subsurface}$
c
{104}-layer 5
Average VE = 6.28 for Fe$_{outmost}$
6.26 for Fe$_{subsurface}$
{104}-layer 5 bond with 10 dissociative H_2O
Average VE = 6.21 for Fe$_{outmost}$
{104}-layer 5 with protonation on bridging O
Average VE = 6.32 for Fe$_{outmost}$
d
{012}-layer 1
Average VE = 6.28
{012}-layer 1 with one O defect
Average VE = 6.37
{012}-layer 1 with two O defects
Average VE = 6.40
Reduced {012} surface (2×1) with half O defects
Average VE = 6.55
{012}-layer 1 bond with 4 H_2O
Average VE = 6.28
{012}-layer 1 bond with 4 dissociative H_2O
Average VE = 6.30
Fe
O
H

Supplementary Figure 14. Bader charge analysis of the facet-exposed Fe. The valence electron (VE) number on the outmost Fe atoms are marked in light yellow, and those on the subsurface Fe atoms are marked in sky blue. The green disks indicate the O vacancies. And the yellow circles highlight the hydroxylation and hydration positions.

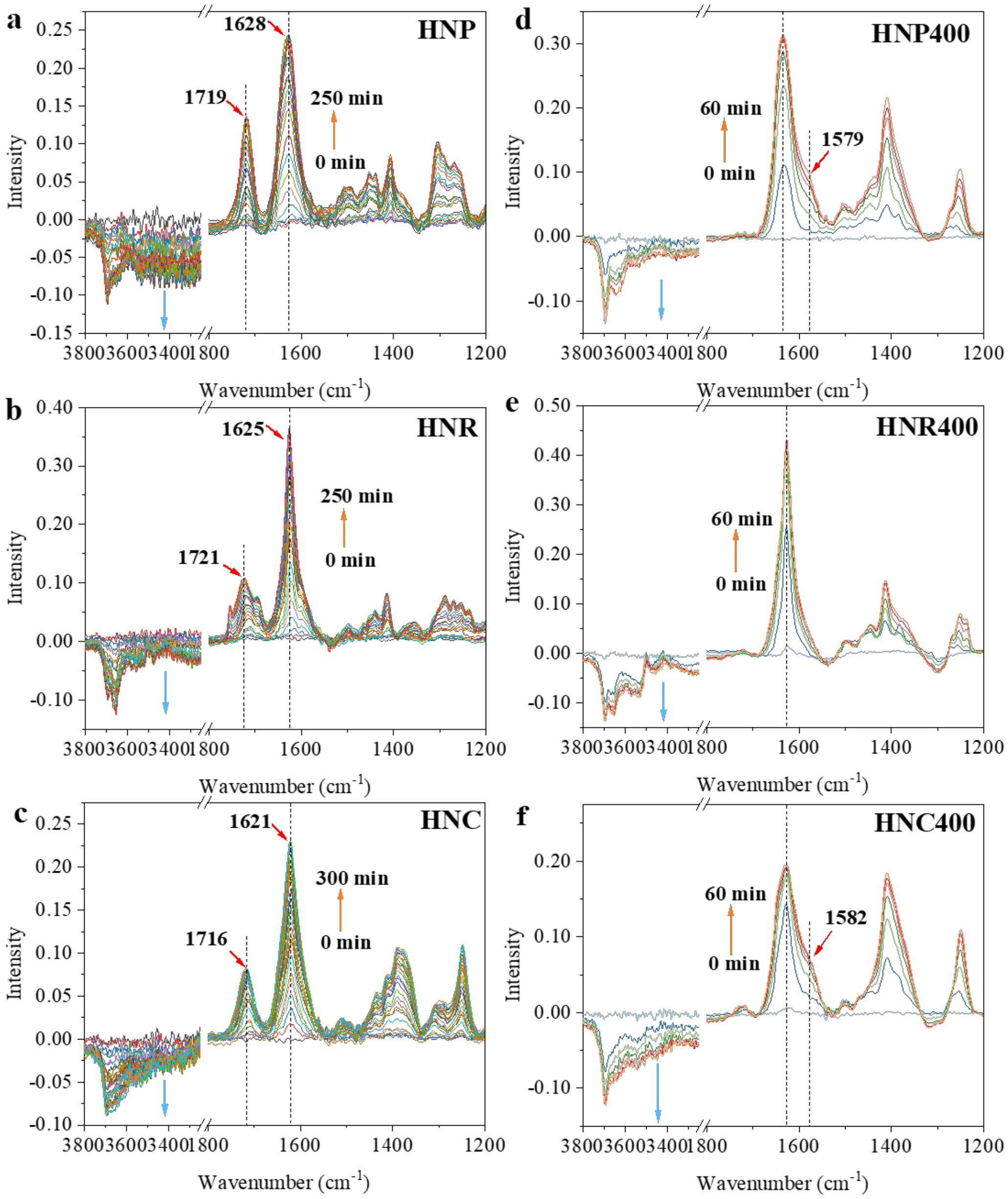

Supplementary Figure 15. *In-situ* DRIFTS measurements for surface Lewis-acid sites. The compound 2-chloro-N,N-dimethylacetamide (Cl-DMA) was used as the probe molecule, as described in our previous study[11]. Cl-DMA was cumulatively adsorbed by the surface active sites on HNP (**a**), HNR (**b**), HNC (**c**), HNP400 (**d**) HNP400, HNR400 (**e**) annd HNC400 (**f**). The hematite of (**d-f**) were pre-calcinated at 400 °C for 2 h. The cumulative adsorption of Cl-DMA was recorded in 10 min interval. The peaks in the red range indicate the Lewis-acid interacted Cl-DMA. The peaks in

the yellow range indicate the physical adsorbed Cl-DMA. The reversal peaks in the blue range indicate that the surface bonded -OH groups and chemisorbed H_2O molecules are substituted by Cl-DMA. Source data are provided as a Source Data file.

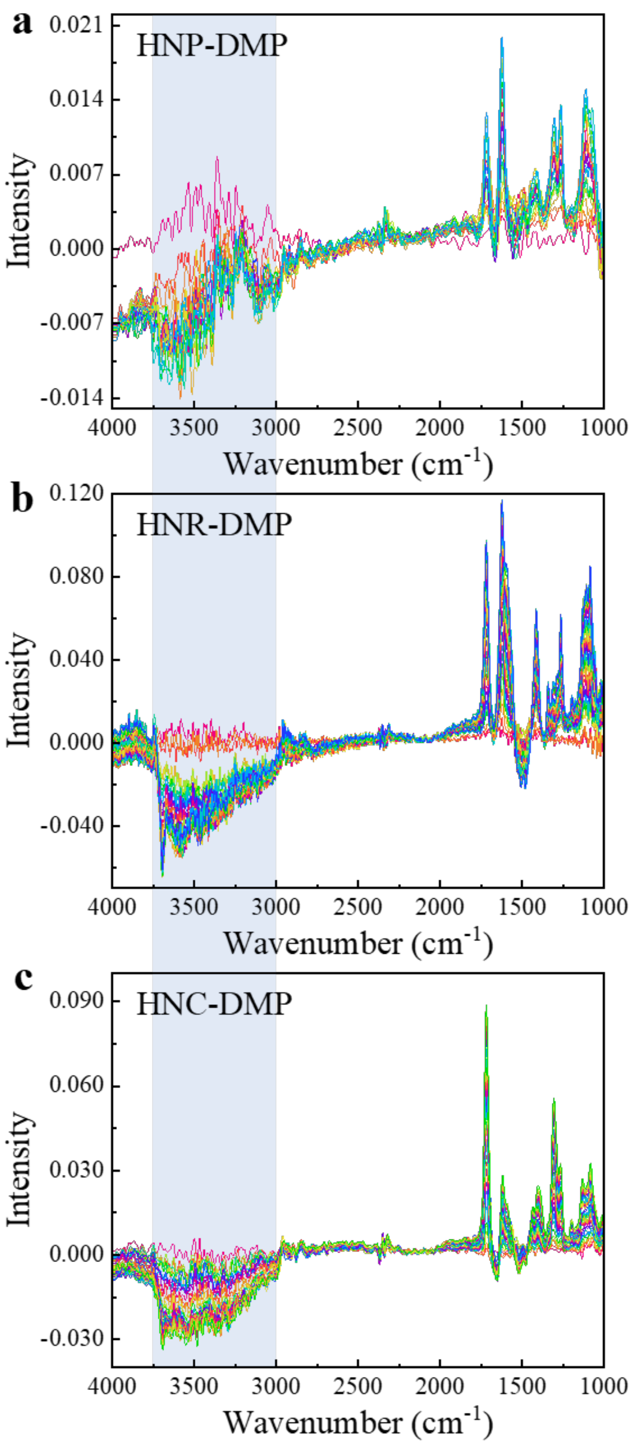

Supplementary Figure 16. *In-situ* DRIFTS measurements by applying gaseous DMP. The blue shading represents the desorption of the surface bonded -OH groups and chemisorbed H_2O molecules, substituted by gaseous DMP. Source data are provided as a Source Data file.

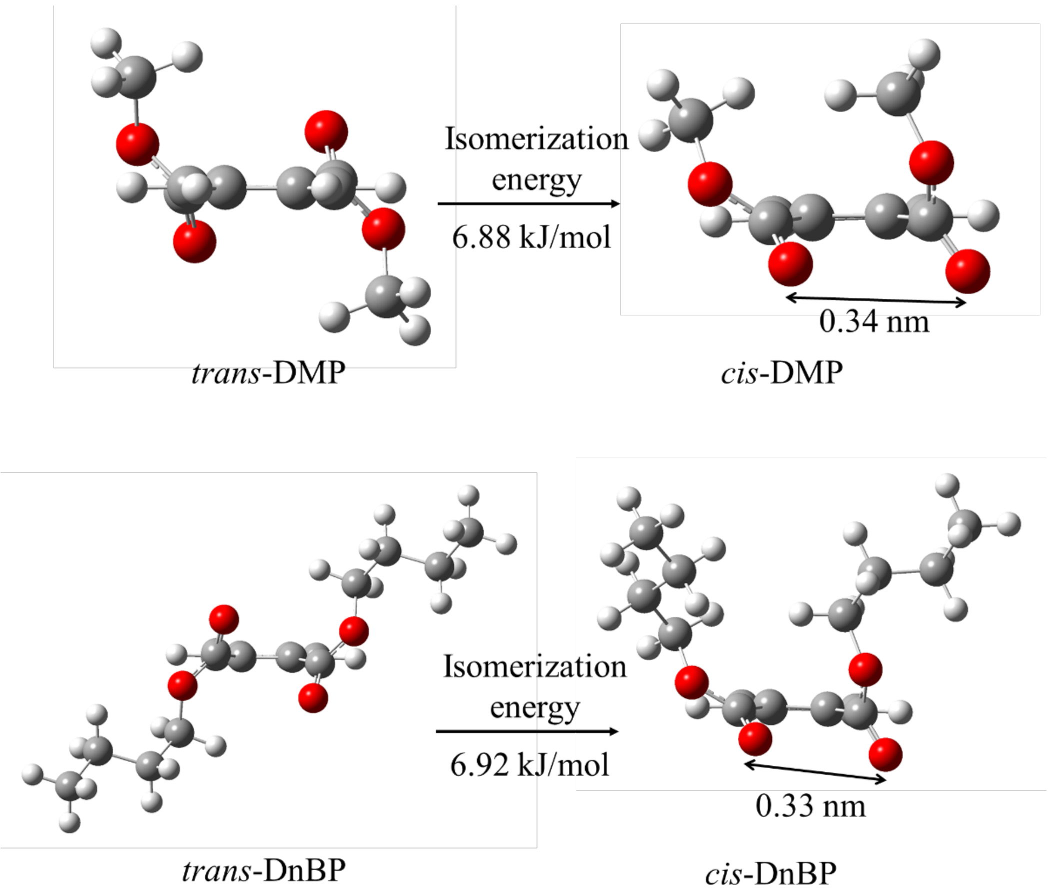

Supplementary Figure 17. Molecule geometries of *trans*-DMP, *cis*-DMP, *trans*-DnBP and *cis*-DnBP. The isomerization energies of *trans*-configuration to *cis*-configuration were obtained.

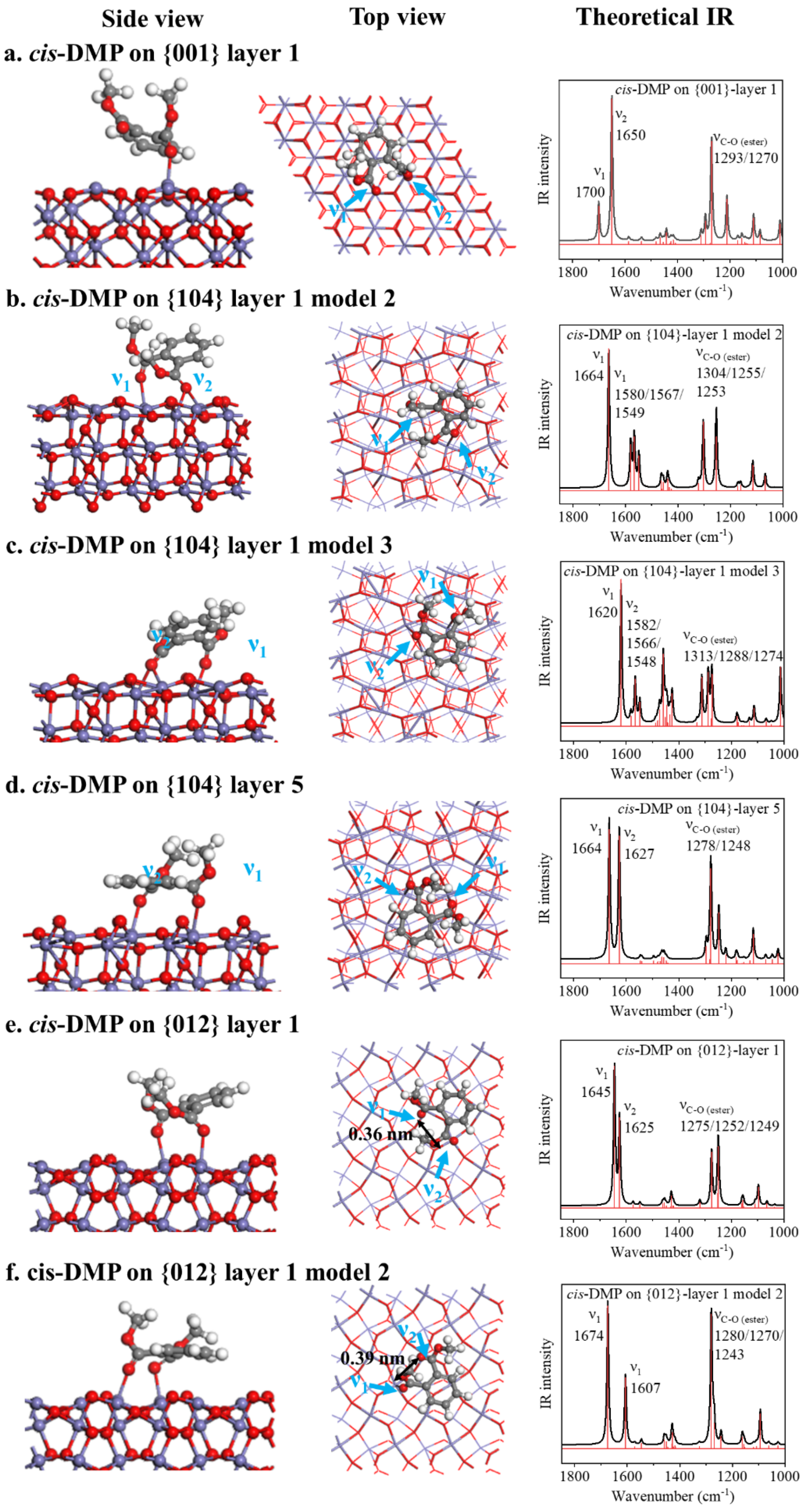

Side view
Top view
Theoretical IR
a. cis-DMP on {001} layer 1
cis-DMP on {001}-layer 1
ν1 1700
ν2 1650
νC-O (ester) 1293/1270
b. cis-DMP on {104} layer 1 model 2
cis-DMP on {104}-layer 1 model 2
ν1 1664
ν1 1580/1567/ 1549
νC-O (ester) 1304/1255/ 1253
c. cis-DMP on {104} layer 1 model 3
cis-DMP on {104}-layer 1 model 3
ν1 1620
ν2 1582/ 1566/ 1548
νC-O (ester) 1313/1288/1274
d. cis-DMP on {104} layer 5
cis-DMP on {104}-layer 5
ν1 1664
ν2 1627
νC-O (ester) 1278/1248
e. cis-DMP on {012} layer 1
0.36 nm
cis-DMP on {012}-layer 1
ν1 1645
ν2 1625
νC-O (ester) 1275/1252/1249
f. cis-DMP on {012} layer 1 model 2
0.39 nm
cis-DMP on {012}-layer 1 model 2
ν1 1674
ν1 1607
νC-O (ester) 1280/1270/ 1243
IR intensity
Wavenumber (cm-1)
1800
1600
1400
1200
1000

Supplementary Figure 18. The adsorption configurations of DMP on {001}, {104} and {012} facets, as well as the corresponding calculated IR spectra. Source data are provided as a Source Data file.

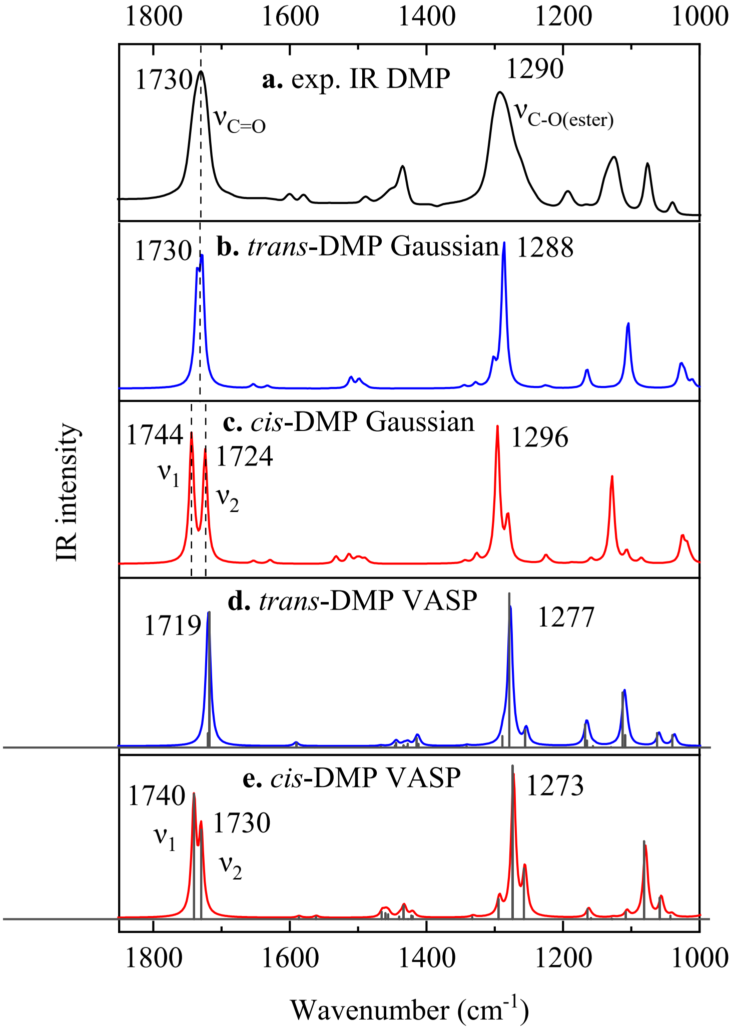

Supplementary Figure 19. The experimental IR spectra of DMP (**a**), and the theoretical IR spectra of *trans*-DMP (**b,d**) and *cis*-DMP (**c, e**) by Gaussian calculation (**b, c**) and VASP calculation (**d, e**). Source data are provided as a Source Data file.

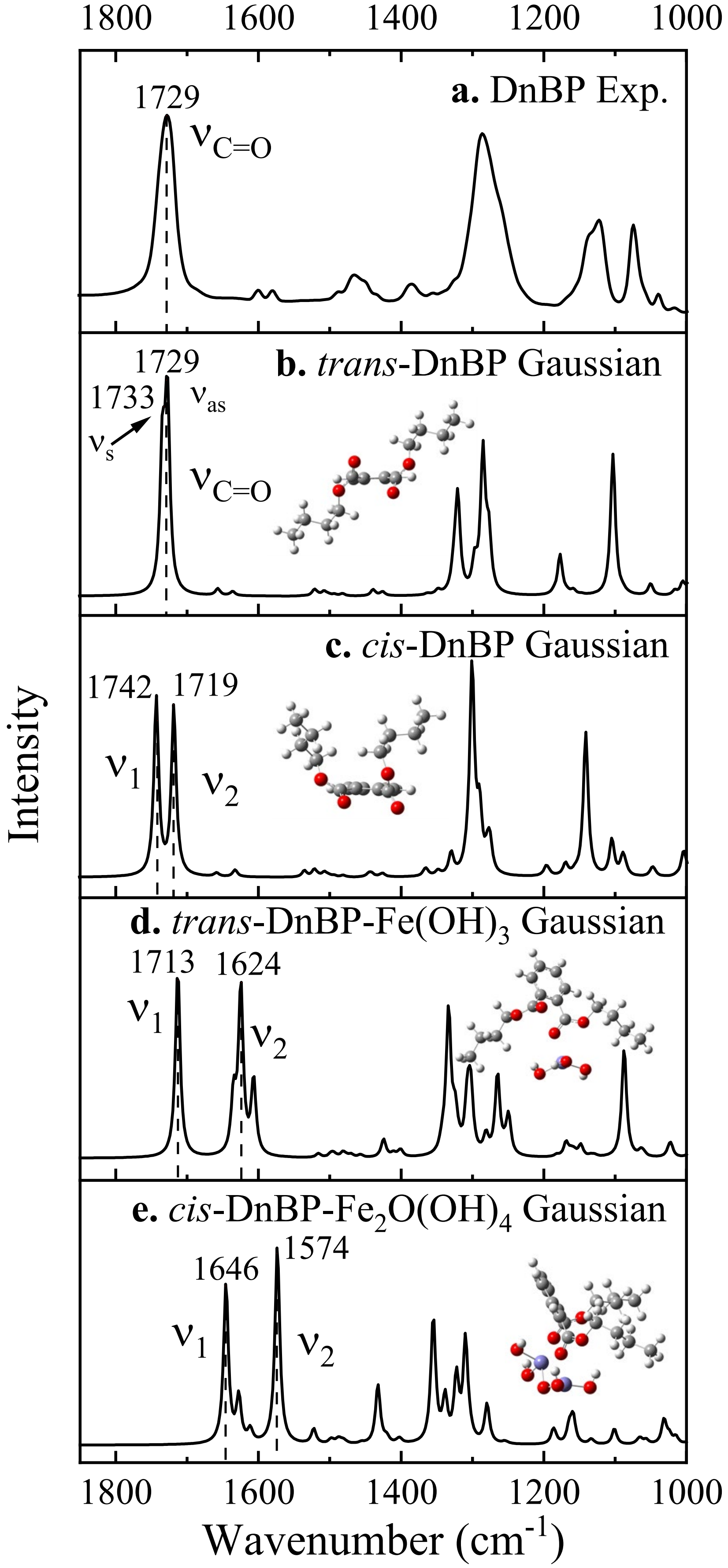

1800
1600
1400
1200
1000
a. DnBP Exp.
1729
ν C=O
b. trans-DnBP Gaussian
1729
1733
ν as
ν s
ν C=O
c. cis-DnBP Gaussian
1742
1719
ν 1
ν 2
d. trans-DnBP-Fe(OH)3 Gaussian
1713
1624
ν 1
ν 2
e. cis-DnBP-Fe2O(OH)4 Gaussian
1646
1574
ν 1
ν 2
Intensity
Wavenumber (cm-1)

368

Supplementary Figure 20. The theoretical IR spectra of *trans-/cis*-DnBP before and after complexing with $Fe(OH)_3$ or $Fe_2O(OH)_4$ clusters. The experimental IR spectrum of DnBP (**a**) was involved for comparison. (**b-e**) The theoretical IR spectra of *trans*-DnBP (**b**), *cis*-DnBP (**c**), *trans*-DnBP monodentate-coordinate with $Fe(OH)_3$ (**d**), and *cis*-DnBP bidentate-coordinate with $Fe_2O(OH)_4$ (**e**). Source data are provided as a Source Data file.

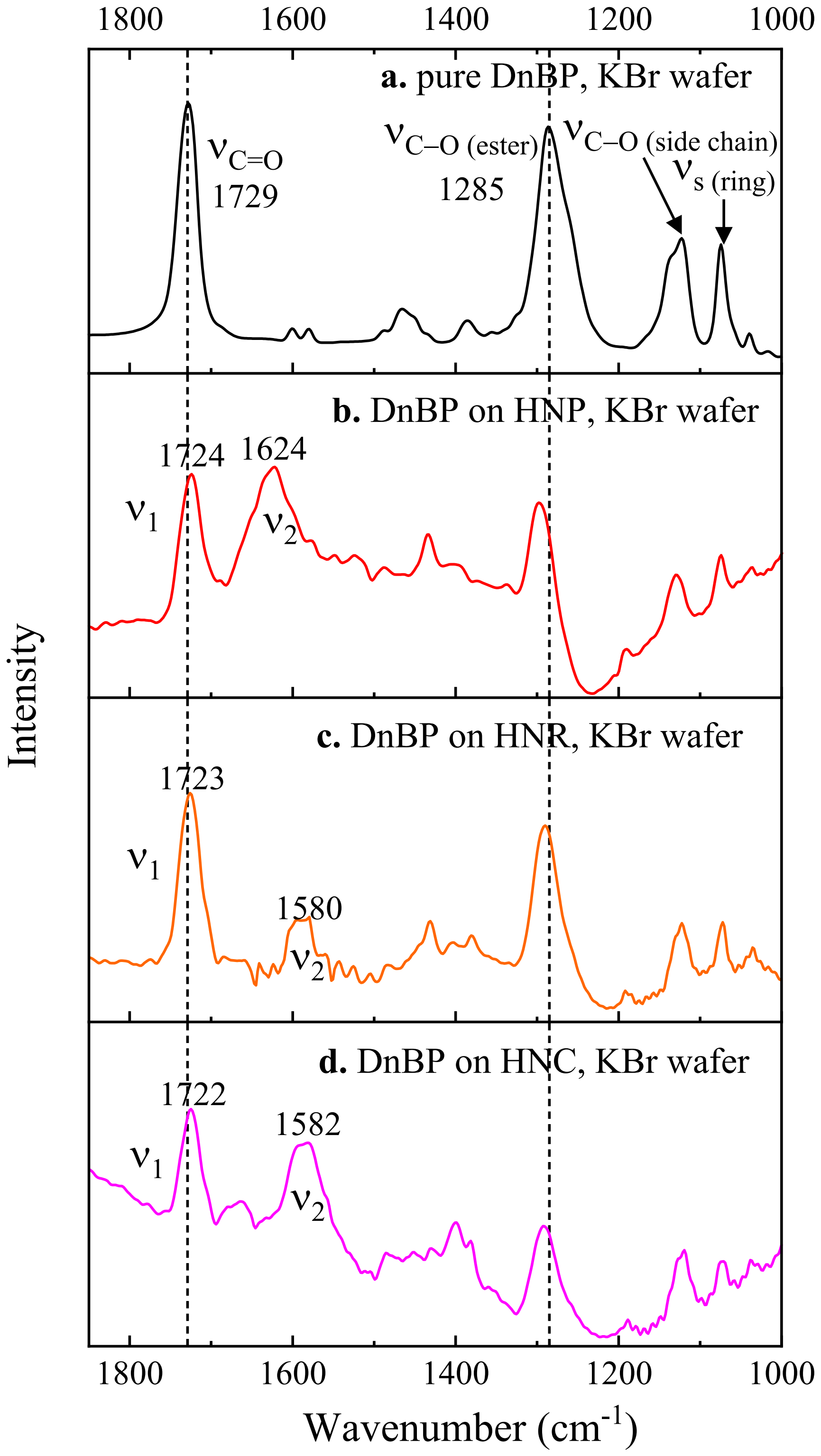

Supplementary Figure 21. The experimental IR spectra of pure DnBP (**a**), and the adsorbed DnBP on HNP (**b**), HNR (**c**), HNC (**d**), by *ex-situ* KBr wafer method. Source data are provided as a Source Data file.

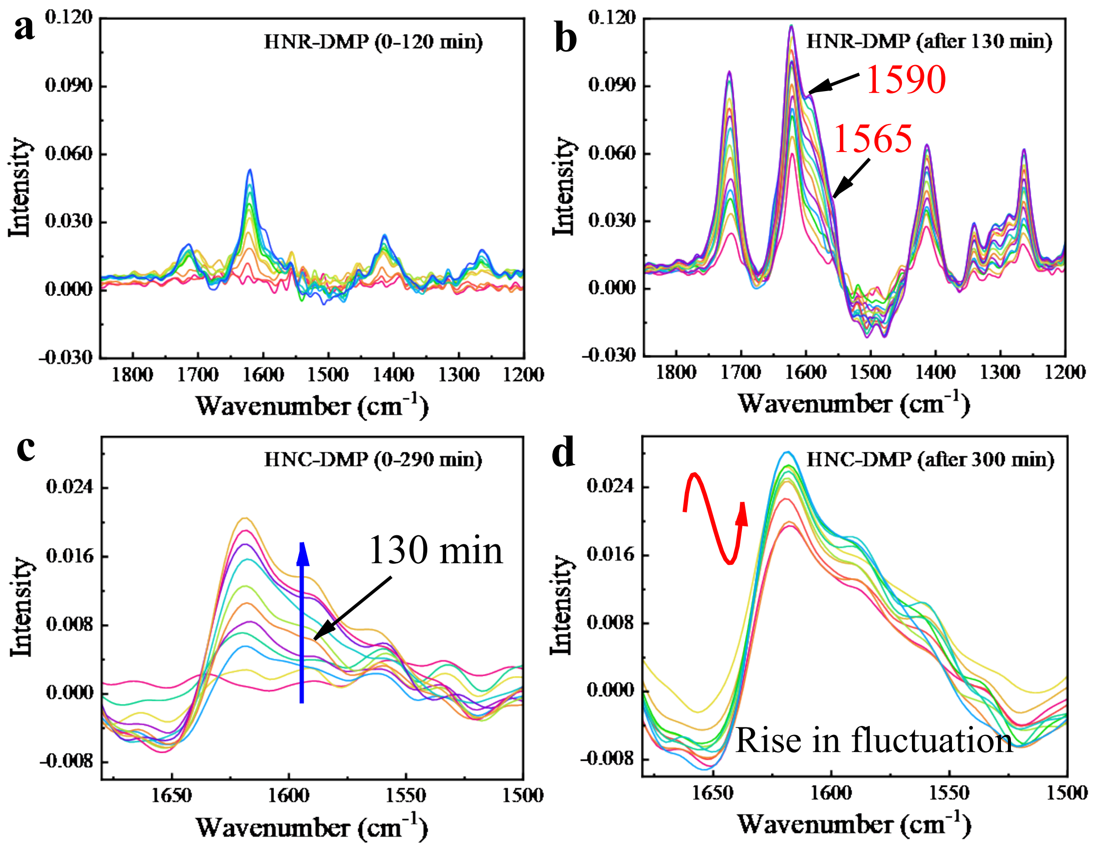

Supplementary Figure 22. The accumulative trend of the carbonyl stretching vibration ($\nu_{C=O}$) at 1565 - 1620 cm^{-1} on HNR (**a, b**) and HNC (**c, d**). The adsorption of DMP on HNC was not accumulated after 300 nm (d), probably due to the rapid consumption of DMP on HNC. Source data are provided as a Source Data file.

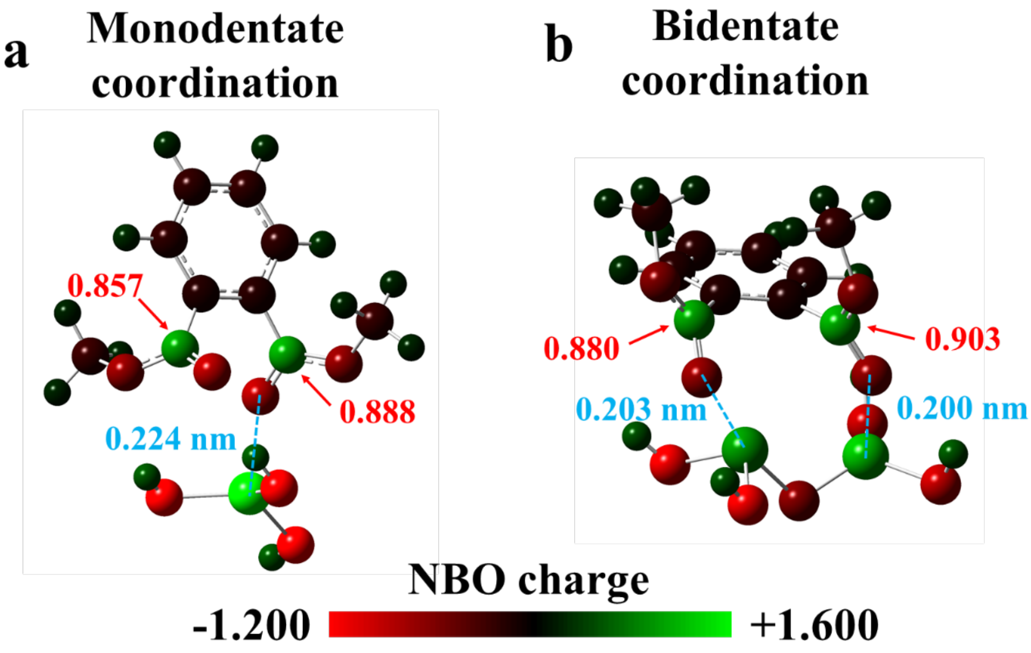

Supplementary Figure 23. The nature bond orbital (NBO) charge distribution and Fe-O bond length of DMP at its monodentate-coordination mode (**a**) and bidentate-coordination mode (**b**).

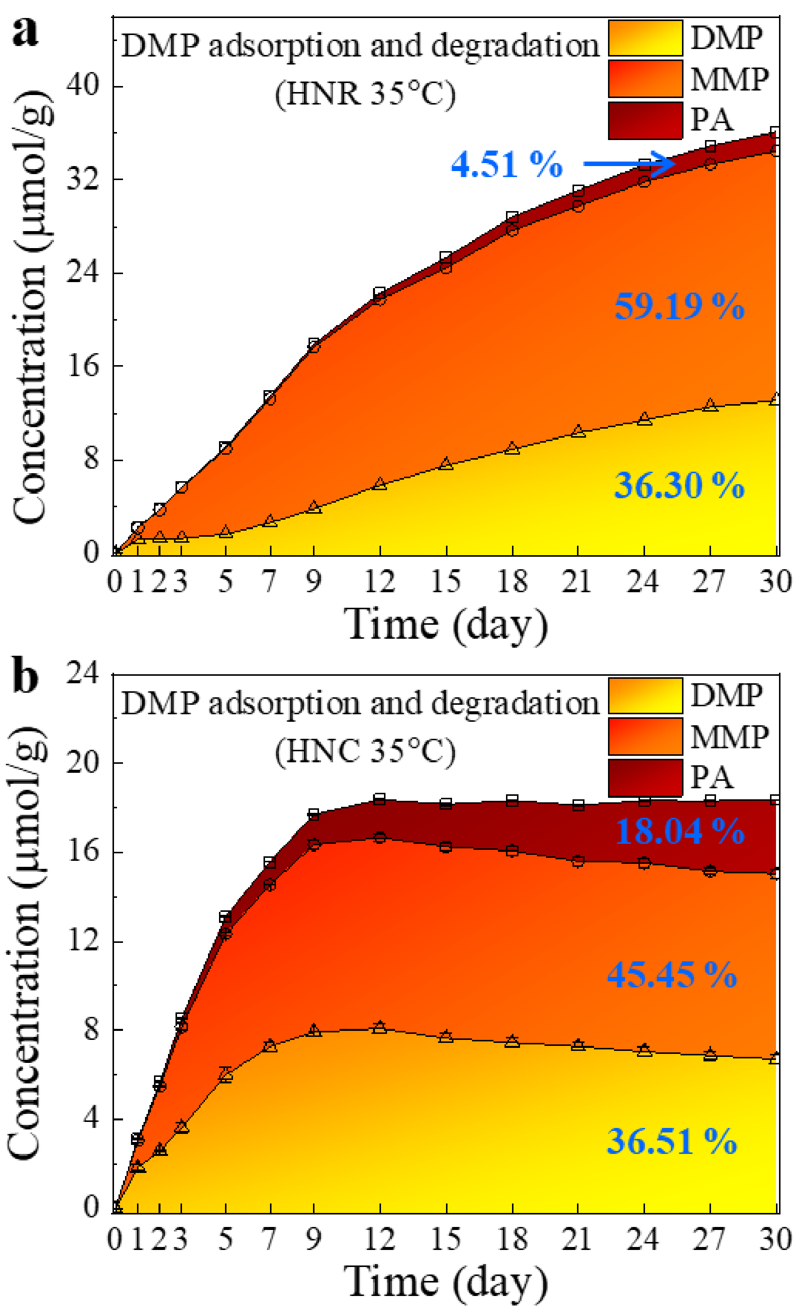

Supplementary Figure 24. The accumulative adsorption of DMP as well as its hydrolytic products (MMP and PA) on HNR (**a**) and HNC (**b**) under RH 76 % and at 35 ˚C. The yellow, orange, and red region represent the cumulative concentration of DMP, MMP, and PA, respectively. Source data are provided as a Source Data file.

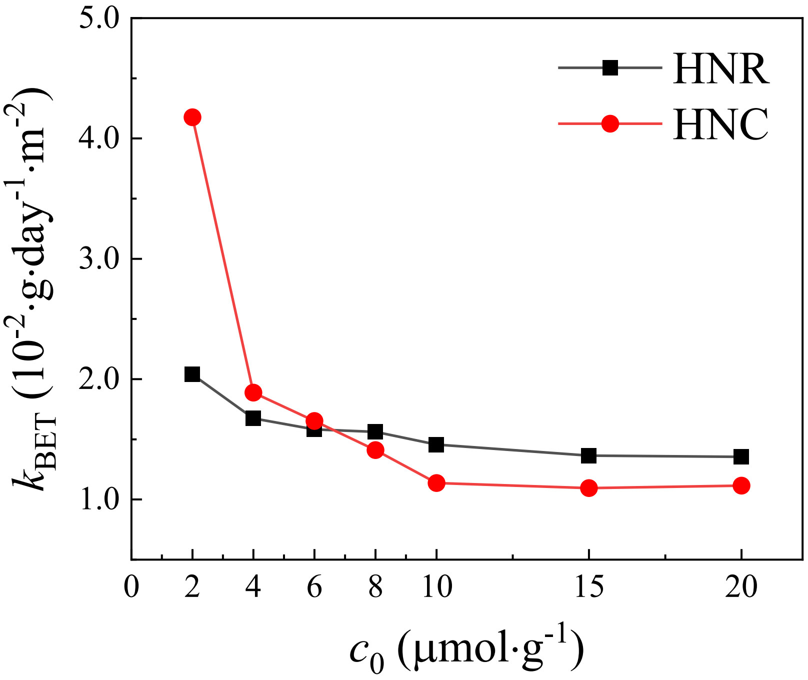

Supplementary Figure 25. The hydrolysis rate constants (k_{BET}) of DMP on HNR and HNC at different initial concentrations (2.0-20.0 μmol·g^{-1}). The experiment was conducted under RH 76%.

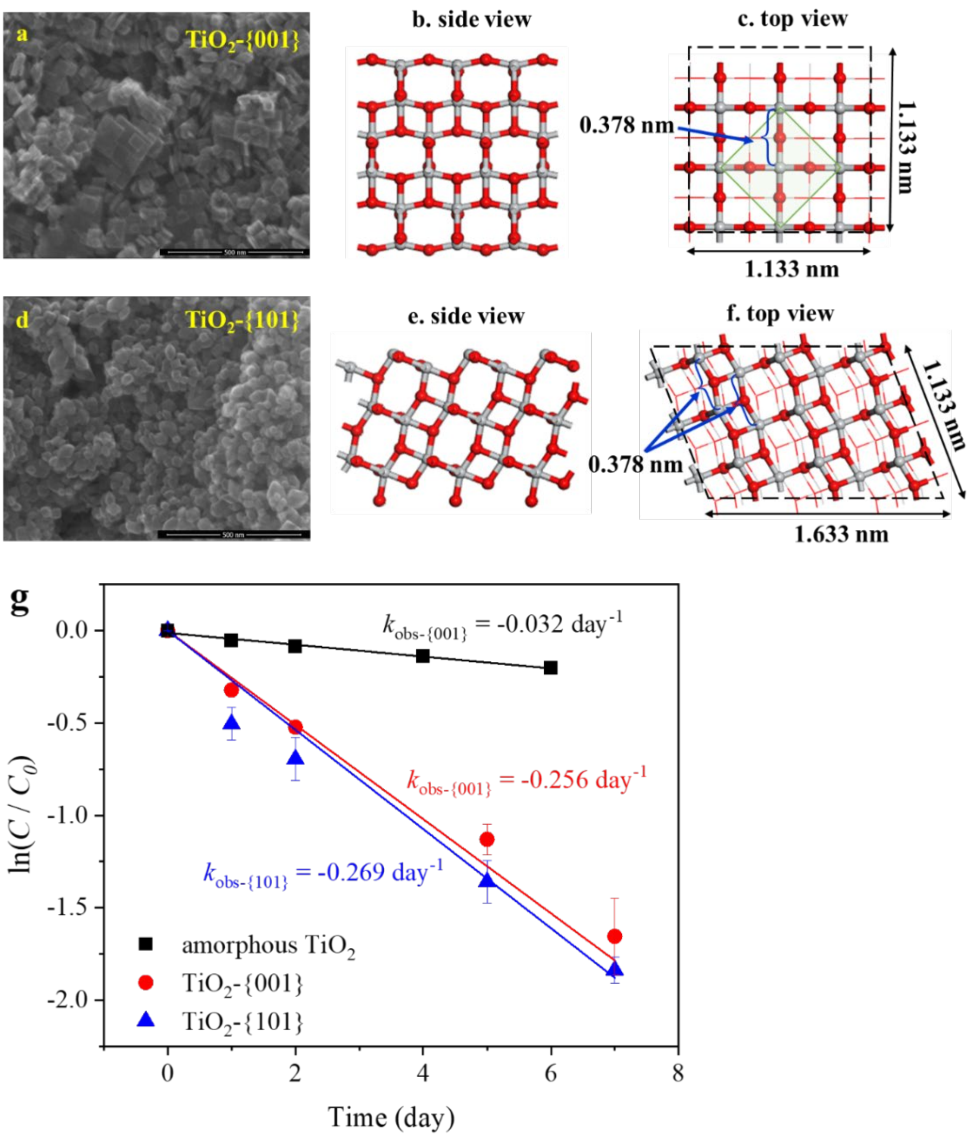

Supplementary Figure 26. Catalytic performance of facet-controlled TiO_2 nanoparticles for hydrolyzing DMP. The scanning electron microscopy (SEM) imagine of synthesized TiO_2-{001} (**a**) and TiO_2-{101} nanoparticles (**d**). The geometric construction of TiO_2-{001} (**b, c**) and TiO_2-{001} (**e, f**) from side view (**b, e**) and top view (**c, f**). The catalytic performance of TiO_2-{001} and TiO_2-{101} to the hydrolysis of DMP (2.0 μmol·g^{-1}) under RH 76%, in comparing with the performance of commercial amorphous TiO_2 nanopowder (**g**). The error bars in g are expressed as the mean value with standard deviations of 2 experimental replicates.

Supplementary Reference:

1. Li, T. et al. Enhanced hydrolysis of p-nitrophenyl phosphate by iron (hydr)oxide nanoparticles: Roles of exposed facets. *Environ. Sci. Technol.* **54**, 8658-8667 (2020).
2. Chatman, S., Zarzycki, P. & Rosso, K.M. Surface potentials of (001), (012), (113) hematite (α-Fe2O3) crystal faces in aqueous solution. *Phys. Chem. Chem. Phys.* **15**, 13911-13921 (2013).
3. Quast, K. The use of zeta potential to investigate the interaction of oleate on hematite. *Miner. Eng.* **85**, 130-137 (2016).
4. Guo, T. et al. Efficient persulfate activation by hematite nanocrystals for degradation of organic pollutants under visible light irradiation: Facet-dependent catalytic performance and degradation mechanism. *Appl. Catal. B* **286**, 119883 (2021).
5. Huang, X., Hou, X., Song, F., Zhao, J. & Zhang, L. Facet-dependent Cr(VI) adsorption of hematite nanocrystals. *Environ. Sci. Technol.* **50**, 1964-1972 (2016).
6. Shen, Z. et al. Facet-dependent adsorption and fractionation of natural organic matter on crystalline metal oxide nanoparticles. *Environ. Sci. Technol.* **54**, 8622-8631 (2020).
7. Schöttner, L. et al. Interaction of water molecules with the α-Fe2O3(0001) surface: A combined experimental and computational study. *J. Phys. Chem. C* **123**, 8324-8335 (2019).
8. Hanioka, N. et al. Hydrolysis of di-n-butyl phthalate, butylbenzyl phthalate and di(2-ethylhexyl) phthalate in human liver microsomes. *Chemosphere* **89**, 1112-1117 (2012).
9. Huang, M. et al. Facet-dependent photoinduced transformation of cadmium sulfide (CdS) nanoparticles. *Environ. Sci. Technol.* **55**, 13132-13141 (2021).
10. Staples, C.A., Peterson, D.R., Parkerton, T.F. & Adams, W.J. The environmental fate of phthalate esters: A literature review. *Chemosphere* **35**, 667-749 (1997).
11. Wu, D. et al. Iron minerals mediated interfacial hydrolysis of chloramphenicol antibiotic under limited moisture conditions. *Environ. Sci. Technol.* **55**, 9569-9578 (2021).
